# Japanese Encephalitis Vaccine Decision Aid for Travelers: A Randomized Clinical Trial

**DOI:** 10.1001/jamanetworkopen.2026.15190

**Published:** 2026-06-01

**Authors:** Sarah L. McGuinness, Owen Eades, Jennifer Morris, Holly Seale, Allen C. Cheng, Karin Leder

**Affiliations:** 1 School of Public Health and Preventive Medicine, Monash University, Melbourne, Victoria, Australia; 2 Department of Infectious Diseases, Alfred Health, Melbourne, Victoria, Australia; 3 Independent Consumer Advisor, Melbourne, Victoria, Australia; 4 School of Population Health, Faculty of Medicine and Health, University of New South Wales, Kensington, New South Wales, Australia; 5 Monash Infectious Diseases Service, Clayton, Victoria, Australia; 6 Victorian Infectious Diseases Service, Royal Melbourne Hospital at the Peter Doherty Institute for Infection and Immunity, Melbourne, Victoria, Australia

## Abstract

**Importance:**

Japanese encephalitis (JE) is a mosquito-borne disease with low infection risk but high consequences. Low uptake of JE vaccines among travelers persists despite effective vaccines. Tools that improve decision quality may help address this gap.

**Objective:**

To evaluate whether a web-based JE vaccine decision aid (JEVaDA) improves decision-making and vaccine uptake among Australian travelers compared with an online government JE resource.

**Design, Setting, and Participants:**

This parallel-group, single-blind randomized clinical trial was conducted online across Australia from November 6, 2024, to July 14, 2025. Adults (aged ≥18 years) planning travel to a JE-endemic country within 6 months were recruited via a research panel.

**Interventions:**

Participants were randomized 1:1 to the JEVaDA intervention, developed to International Patient Decision Aid Standards, or an online government JE resource (the active comparator).

**Main Outcomes and Measures:**

The primary outcome was postintervention decisional conflict, measured using the Decisional Conflict Scale (DCS) (scores range from 0 to 100, with higher scores indicating greater conflict). Secondary outcomes included change in JE knowledge, intention to vaccinate, and self-reported vaccine uptake. Analyses used regression models adjusted for baseline values, age, and gender.

**Results:**

Of the 1879 individuals screened, 814 were randomized and 769 completed preintervention and postintervention assessments (modified intention-to-treat population: 373 in the intervention group and 396 in the comparator group). Their mean (SD) age was 44.7 (15.2) years; 394 (51.2%) identified as women. The intervention and comparator groups showed significant reductions in decisional conflict (mean DCS score change, −10.94 [95% CI, −12.81 to −9.07] vs −11.58 [−13.24 to −9.91] points, respectively; both *P* < .001), with no between-group difference (β, −0.87 [95% CI, −2.93 to 1.19]; *P* = .41). JE knowledge improved in both groups (intervention vs comparator: 2.27 [95% CI, 2.00-2.54] vs 2.63 [95% CI, 2.36-2.91] correct responses; *P* < .001), with no between-group difference (incidence rate ratio [IRR], 0.95 [95% CI, 0.90-1.00]; *P* = .07). Positive change in intention to vaccinate occurred in 72 participants (19.3% [95% CI, 1.53%-2.33%]) in the intervention group vs 66 (16.7% [95% CI, 1.30%-2.04%]) in the comparator group (adjusted odds ratio [AOR], 1.19 [95% CI, 0.80-1.76]; *P* = .40). Among the 348 travelers completing follow-up after travel to JE risk areas, vaccine uptake was 35.1% overall (n = 122) and was significantly higher in the intervention group than in the comparator group (69 of 161 [42.9%] vs 53 of 187 [28.3%]; AOR, 2.22 [95% CI, 1.36-3.61]; *P* = .001).

**Conclusions and Relevance:**

In this randomized clinical trial, the web-based JEVaDA was not associated with a further reduction in decisional conflict compared with an active comparator but was associated with higher vaccine uptake. These findings suggest that decision aids can support informed, values-congruent choices in complex, preference-sensitive health decisions such as travel vaccination and beyond.

**Trial Registration:**

anzctr.org.au Identifier: ACTRN12624001176550

## Key Points

**Question:**

Can a web-based Japanese encephalitis (JE) vaccine decision aid improve decision-making and vaccine uptake among Australian travelers compared with an online government JE information resource?

**Findings:**

In this randomized clinical trial of 814 adults planning travel to JE-endemic countries, both resources significantly reduced decisional conflict, with no between-group difference. Among the 348 travelers completing follow-up, vaccine uptake was significantly higher in the intervention group (43% vs 28%).

**Meaning:**

Decision aids may support informed, values-congruent choices in complex, preference-sensitive health decisions such as travel vaccination, complementing clinician advice.

## Teaser

This randomized clinical trial evaluates whether a web-based Japanese encephalitis vaccine decision aid improves vaccine decision-making and uptake among Australian travelers.

## Introduction

Japanese encephalitis (JE) is a mosquito-borne viral disease endemic across much of Southeast Asia and the Western Pacific.^1^ Although most cases of JE infection are asymptomatic, the fatality rate is 14% to 26% among reported symptomatic cases, and nearly half of survivors experience long-term neurologic sequelae.^2,3^ Childhood vaccination campaigns have reduced incidence in endemic countries, yet transmission persists via enzootic cycles involving mosquitoes and animal hosts.^4,5,6,7^ Risk is expanding geographically, including in Australia.^8,9^ For travelers, absolute risk is low but varies by season, destination, and activities and may accrue across repeated trips.^10,11^ Fatal cases of JE have occurred in short-term travelers.^10^

Two safe, effective JE vaccines are available in Australia, but uptake among travelers remains low.^12,13,14,15,16^ National guidelines recommend vaccination for individuals with a trip duration of 1 month or longer in endemic areas during the transmission season, with case-by-case consideration for shorter stays.^17^ These nuanced recommendations, combined with high vaccine cost and the low-probability but high-consequence nature of JE, create a complex decision-making environment for travelers and clinicians.^18,19^

Decision aids are tools designed to help people participate in decision-making about health care options, including vaccines. At a minimum, decision aids describe the health condition or problem; make explicit the decision; provide information on options, risks, and benefits; and help patients clarify which risks and benefits matter most to them.^20^ Decision aids can improve knowledge, reduce decisional conflict, and support informed choices.^21,22^ Benefits are greatest for preference-sensitive decisions, where choices depend on individual values and circumstances.^23^ Despite this, decision aids have not been evaluated for travel vaccines. A previous survey of Australian travelers found strong interest in web-based, interactive decision aids.^24^ Building on these insights, we codesigned and user tested a web-based JE vaccine decision aid (JEVaDA) with travelers and clinicians to optimize clarity, usability, and relevance.^25^ This randomized clinical trial evaluated the impact of the JEVaDA on informed vaccine decision-making and vaccine uptake among Australian travelers, compared with an online government JE information resource.

## Methods

### Trial Design, Setting, and Participants

We conducted a parallel-group, single-blind, individually randomized clinical trial online across Australia. This study used a pragmatic design, meaning it evaluated the effectiveness of the intervention compared with a standard government fact sheet under conditions reflecting real-world exposure to health information in a travel decision-making context. The study was approved by the Monash University Human Research Ethics Committee. The trial protocol and the statistical analysis plan are provided in Supplement 1. No protocol changes occurred after study commencement. Participants provided informed consent electronically via an “I agree” button on the study landing page. The study followed the Consolidated Standards of Reporting Trials (CONSORT) reporting guideline.

Eligible participants were adults (aged ≥18 years) residing in Australia, planning overseas travel to a JE-endemic country within 6 months, and able to complete English-language surveys. Participants were recruited via a research-only consumer panel (Online Research Unit [ORU]) using stratified sampling for demographic representation. Individual member data were not shared with researchers. Panel members were incentivized through ORU’s point-cash system.

### Intervention Development

A consumer representative and clinicians with travel medicine and vaccination expertise formed the steering group. Codesign and user testing with 25 consumers and 13 clinicians informed decision aid content, usability, and outcome selection.^25^

### Intervention and Comparator

The intervention was a web-based JEVaDA^26^ developed to International Patient Decision Aids Standards (IPDAS)^27^ through a multistep codesign process.^25,28^ The decision aid provided disease and vaccine information, decision-making prompts, and a values clarification exercise with automated feedback based on a coded summary score from the 7 items (eFigure 1 in Supplement 2), as well as a short-form PDF, FAQs, and traveler stories. The active comparator was an online government JE information resource.^29^ Both resources were evidence based and publicly accessible; their content was similar (eTable 1 in Supplement 2). Readability was assessed using the Simple Measure of Gobbledegook (SMOG) index following an established approach.^30^

### Procedures

Data were collected via secure REDCap (research electronic data capture) surveys.^31,32^ ORU invitations directed participants to a Monash University–hosted landing page containing the explanatory statement, eligibility screening questions, and electronic consent. Eligible participants then completed a preintervention survey capturing demographics, travel plans, health status, JE vaccination history, and baseline outcomes, including decisional conflict. General vaccine attitudes were assessed using the 4-item Vaccine Confidence Index (VCI).^33^ Participants next received a link to their allocated resource and reviewed it at their own pace before completing the postintervention survey assessing postintervention outcomes and engagement. Those opting into follow-up were contacted approximately 2 months after their planned trip to report JE vaccine uptake and travel behaviors.

Engagement was measured via self-report in both groups (extent of review, time spent, and resource identification). REDCap time stamps provided an additional estimate of time spent reviewing the allocated resource. Website analytics were only available for the decision aid; visits during the study period were assumed to represent participants.

### Outcomes

The primary outcome was postintervention decisional conflict, measured using the 16-item Decisional Conflict Scale (DCS), adjusted for baseline DCS score, age, and gender.^34^ The DCS assesses decision quality across 5 subscales: informed, values clarity, support, uncertainty, and effective decision.^35^ Items use a 5-point Likert scale (ranging from 0 for strongly agree to 4 for strongly disagree), converted to a score ranging from 0 to 100 (with higher scores reflecting greater conflict). Scores less than 25 are associated with implementing decisions, while scores greater than 37.5 reflect uncertainty or delay.^34^

Secondary outcomes were change in JE knowledge, intention to vaccinate, and self-reported JE vaccine uptake. Knowledge was measured as the change in correct responses to 9 items (“true,” “false,” or “not sure”) from before to after intervention (eTable 2 in Supplement 2). Intention was measured by changes in response to this question: “Do you want to get a JE vaccine before your upcoming trip?” (with response options of “no,” “yes,” “not sure,” or “already vaccinated”), adapted from the World Health Organization Behavioral and Social Drivers of Vaccination survey.^36^ Uptake was assessed in the follow-up survey via this question: “Did you receive a vaccine for JE before your trip?” (with response options of “yes,” “no,” or “I don’t remember”). No adverse events were anticipated or assessed, as both interventions were information resources.

#### Sample Size

The target sample size was based on detecting an effect size of 0.30 for the primary outcome per the DCS user manual.^37^ Using analysis of covariance (ANCOVA) assumptions (within-participant correlation between preintervention and postintervention DCS scores of 0.5; 70% completion), approximately 500 participants were required to achieve greater than 90% power (α = .05).

#### Randomization

Participants were randomized 1:1 to the JEVaDA intervention or the active comparator, stratified by age, gender, and jurisdiction. Allocation was automated in REDCap using a sequence generated by an independent statistician. Participants were not blinded; the primary analyst (S.L.M.) was blinded to group labels via coded identifiers.

### Statistical Analysis

Analyses were conducted in Stata, version 18 (StataCorp LLC), using a modified intention-to-treat population (participants with complete preintervention and postintervention DCS scores). Baseline characteristics are reported as means (SDs) or frequencies (percentages).

The primary outcome (postintervention DCS score) was modeled using ANCOVA with group allocation as the main predictor and baseline DCS score, age, and gender as covariates; subscales were analyzed similarly. A post hoc sensitivity analysis excluded extreme outliers identified on inspection of a QQ plot of model residuals; no numerical cutoff was prespecified. Robustness to missing data was assessed using multiple imputation by chained equations with predictive mean matching (20 imputed datasets), including ANCOVA covariates (intervention group, baseline DCS score, age, and gender) and auxiliary baseline variables (education, vaccination intention, and knowledge). Results are reported as regression coefficients (β values) with 95% CIs and *P* values. Two-tailed *P* < .05 was considered significant.

Secondary outcomes used regression models appropriate to data type. Knowledge was analyzed using Poisson regression, adjusting for baseline knowledge, age, and gender; results are presented as incidence rate ratios (IRRs) with 95% CIs. Overdispersion was examined with a negative-binomial model. Intention and uptake were analyzed using logistic regression with relevant covariates. Findings are reported as adjusted odds ratios (AORs) with 95% CIs. Missing data were minimal (one age value imputed to mean).

## Results

**Figure 1.**
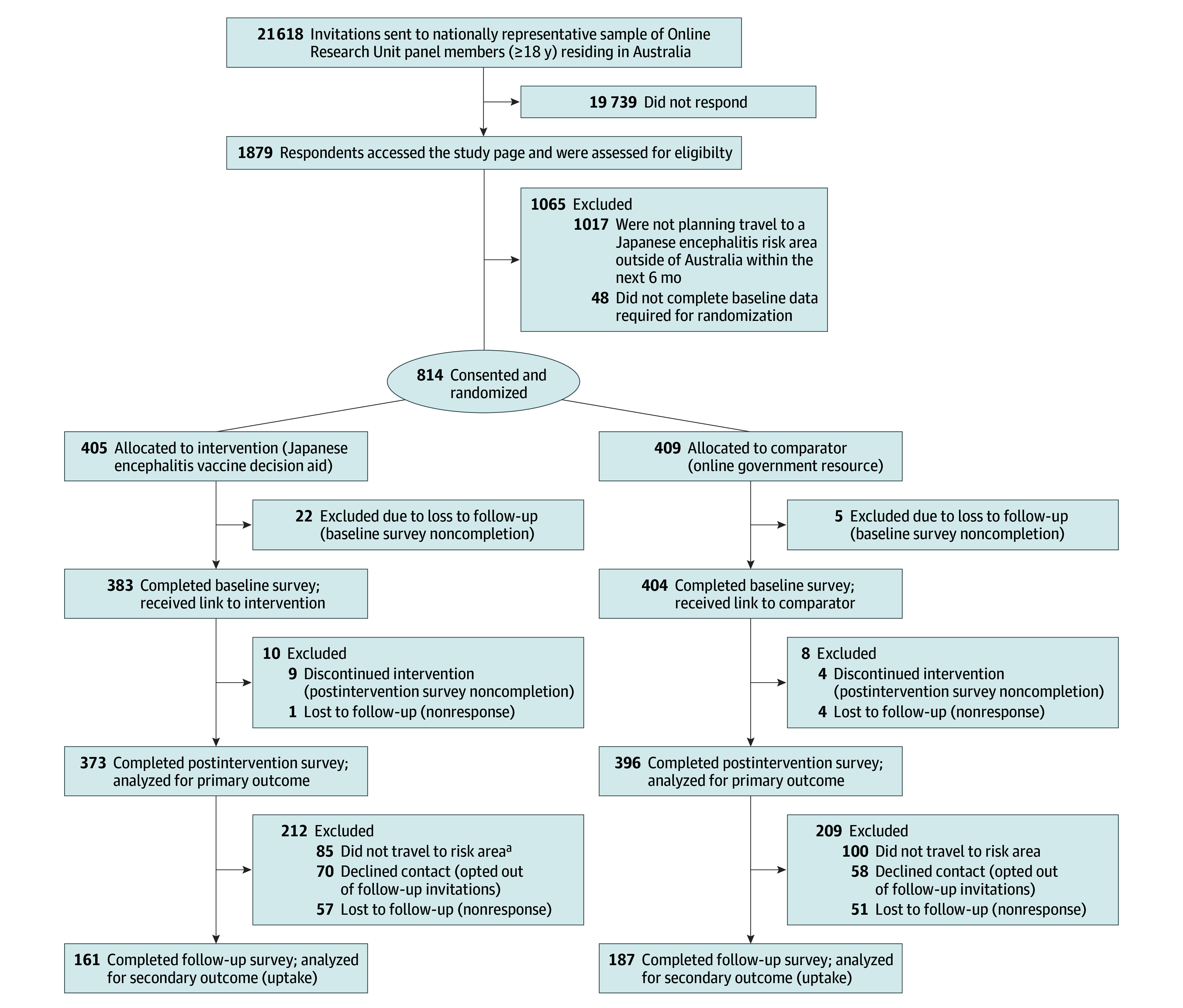
CONSORT Diagram of Participant Flow Through the Trial Participants were recruited via a research-only consumer panel (the Online Research Unit) using a randomly stratified sampling approach to ensure national representativeness based on age, sex, and geographic location. ^a^Participants who completed follow-up but did not travel to a Japanese encephalitis risk destination were excluded from uptake analysis because the vaccination decision was no longer relevant.

We screened 1879 participants between November 6 and 10, 2024 (Figure 1). Of these, 814 consented and were randomized, and 769 were included in the primary analysis (373 in the intervention group and 396 in the comparator group) based on availability of postintervention DCS scores. Planned travel dates spanned November 2024 to May 2025. The groups were similar at baseline (Table 1). Their mean (SD) age was 44.7 (15.2) years (range, 19-93 years); 394 (51.2%) identified as women and 373 (48.5%) identified as men. Most participants were Australian born (479 [62.3%]) and spoke only English at home (571 [74.3%]). Two-thirds (524 [68.1%]) held a university degree, and 104 (13.5%) indicated they may need support with health literacy.

**Table 1.** Baseline Demographic and Travel Characteristics of the Modified Intention-to-Treat Population^a^

Characteristic	Comparator group (online government resource) (n = 396)	Intervention group (JEVaDA tool) (n = 373)	All participants (N = 769)
Age, mean (SD), y	44.8 (15.2)	44.6 (15.2)	44.7 (15.2)
Gender			
Man	194 (49.0)	179 (48.0)	373 (48.5)
Woman	201 (50.8)	193 (51.7)	394 (51.2)
Nonbinary	1 (0.3)	1 (0.3)	2 (0.3)
Born in Australia	234 (59.1)	245 (65.7)	479 (62.3)
Spoke only English at home	287 (72.5)	284 (76.1)	571 (74.3)
University degree	274 (69.2)	250 (67.0)	524 (68.1)
May need support with health literacy^b^	54 (13.6)	50 (13.4)	104 (13.5)
Main travel destination			
Japan	79 (19.9)	83 (22.3)	162 (21.1)
Singapore	41 (10.4)	46 (12.3)	87 (11.3)
Indonesia	42 (10.6)	43 (11.5)	85 (11.1)
Thailand	34 (8.6)	28 (7.5)	62 (8.1)
China	38 (9.6)	30 (8.0)	68 (8.8)
India	35 (8.8)	30 (8.0)	65 (8.5)
Other	127 (32.1)	113 (30.3)	240 (31.2)
Trip duration ≥1 mo	85 (21.5)	81 (21.7)	166 (21.6)
Mostly urban travel	241 (60.9)	226 (60.6)	467 (60.7)
Previous travel to Asia	294 (74.2)	262 (70.2)	556 (72.3)
Previous travel to visit friends or relatives^c^	186 (47.0)	169 (45.3)	355 (46.2)
Heard of JE before	140 (35.4)	131 (35.1)	271 (35.2)
Previous JE vaccination	27 (6.8)	40 (10.7)	67 (8.7)

Abbreviations: JE, Japanese encephalitis; JEVaDA, Japanese encephalitis vaccine decision aid.

^a^ Unless indicated otherwise, values are presented as No. (%) of participants.

^b^ Responded “sometimes,” “often,” or “always” to the single-item literacy screener.

^c^ Asked only of participants who reported previous overseas travel.

Common destinations among participants included Japan (162 [21.1%]), Singapore (87 [11.3%]), Indonesia (85 [11.1%]), and Thailand (62 [8.1%]). A total of 1 in 5 participants (166 [21.6%]) planned a trip duration of 1 month or longer, and 467 (60.7%) planned mostly urban travel. Prior travel to Asia was common (556 [72.3%]), and 355 participants (46.2%) reported previous travel to visit friends or relatives. Awareness of JE was low (271 [35.2%]), and prior JE vaccination was uncommon (intervention vs comparator: 40 [10.7%] vs 27 [6.8%]). Baseline vaccine confidence was high and similar between groups, with more than 80% agreeing with each of the 4 VCI statements—that vaccines are important (660 [85.8%]), safe (623 [81.0%]), effective (669 [87.0%]), and compatible with their beliefs (653 [84.9%]) (eTable 3 in Supplement 2).

### Resource Engagement

After the intervention, 525 participants (68.3%) reported reviewing their allocated resource in full, 201 (26.1%) reviewed part, and 43 (5.6%) reviewed none; proportions were similar across groups. JEVaDA web analytics recorded 198 unique user visits (>95% via direct links from Australia), with 103 users completing all steps; the bounce rate (users leaving after viewing only 1 page) was less than 5% (eTable 4 in Supplement 2). Because cookie-based tracking only records visits where cookies are accepted, this represents only partial capture of total traffic. Analytics were not available for the comparator website. Most participants (554 [72.0%]) correctly identified their assigned resource (intervention vs comparator: 293 [78.6%] vs 261 [65.9%]). Participants estimated spending 11 minutes reviewing resources (median, 10 [IQR, 5-13] minutes in both groups); REDCap time stamps suggested a mean (SD) of 8 (7.4) minutes spent (intervention vs comparator: 8.1 [7.5] minutes vs 7.6 [7.4] minutes). The median SMOG readability scores were 9.2 (IQR, 8.9-10.1) for the intervention group and 10.5 (IQR, not available) for the comparator group, both above the recommended grade 8 reading level for public-facing patient information.

### Primary Outcome: Decisional Conflict

**Figure 2.**
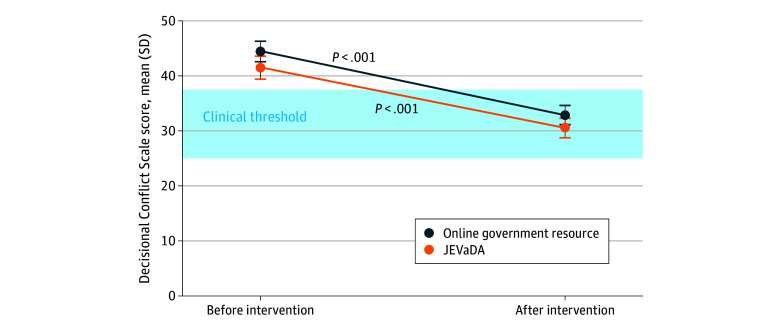
Line Graph of Decisional Conflict Scale (DCS) Scores Among the Intervention and Comparator Groups Before and After Review of Materials The intervention group received the Japanese encephalitis vaccine decision aid (JEVaDA); the comparator group received the online government resource. Error bars indicate 95% CIs for unadjusted means. *P* values are from analysis of covariance models for within-group change. Horizontal dashed lines mark clinical thresholds: scores below 25 were associated with implementing decisions, and scores above 37.5 were associated with uncertainty or delay. The intervention group had a slightly lower baseline mean score.

Both groups showed large reductions in decisional conflict (Figure 2). The mean reduction in DCS score was −10.94 (95% CI, −12.81 to −9.07) points in the intervention group and −11.58 (95% CI, −13.24 to −9.91) points in the comparator group (both *P* < .001). Overall, 515 of 769 participants (67.0%) experienced a reduction in decisional conflict (eFigure 2 in Supplement 2). A total of 181 participants (23.5%) had postintervention DCS scores below 25, the threshold associated with decision implementation, with similar proportions in the intervention (91 [24.4%]) and comparator (90 [22.7%]) groups. Conversely, 238 participants (30.9%) had postintervention DCS scores remaining greater than 37.5, the level linked to uncertainty or delay, with fewer in the intervention group (102 [27.4%]) than in the comparator group (136 [34.3%]).

In the primary ANCOVA, adjusted postintervention DCS scores did not differ significantly between groups (β, –0.87 [95% CI, −2.93 to 1.19]; *P* = .41) (Table 2). The adjusted standardized effect size was small (Cohen *d*, –0.06 [95% CI, –0.20 to 0.08]), indicating a negligible between-group difference. Higher baseline DCS scores predicted higher postintervention scores (β, 0.49 [95% CI, 0.44-0.54]; *P* < .001), and older age predicted modest reductions in DCS scores (β, –0.12 [95% CI, −0.19 to −0.05]; *P* = .001); gender was not associated. A sensitivity analysis excluding 6 extreme outliers yielded similar results (β, –1.52 [95% CI, –3.44 to 0.41]; *P* = .12) (eTable 5 in Supplement 2). Multiple imputation produced a similar estimate (β, –0.96 [95% CI, –3.01 to 1.08]; *P* = .36), and results were unchanged when auxiliary variables were excluded (β, –0.98 [95% CI, –3.02 to 1.05]; *P* = .34). Both groups’ scores improved across all 5 DCS subscales, with no significant between-group differences (eTables 6 and 7 in Supplement 2).

**Table 2.** Primary Analysis of Covariance Model Prediction of Postreview DCS Score^a^

Variable	Change in DCS score, β coefficient (95% CI)^b^	*P* value
Group		
Comparator (online government resource) vs intervention (JEVaDA [reference])	−0.87 (−2.93 to 1.19)	.41
Covariate		
Baseline DCS score (per 1 point)	0.49 (0.44-0.54)	<.001
Age (continuous)	−0.12 (−0.19 to −0.05)	.001
Gender (women compared with men)	−1.59 (−3.66 to 0.49)	.13

Abbreviations: DCS, Decisional Conflict Scale; JEVaDA, Japanese encephalitis vaccine decision aid.

^a^ The DCS is scored from 0 to 100, with higher scores indicating greater conflict.

^b^ Negative β coefficients indicate lower adjusted postreview DCS scores. The model adjusted for baseline DCS score, age, and gender.

### Secondary Outcomes

#### Knowledge of JE

JE knowledge improved significantly in both groups (intervention vs comparator: 2.27 [95% CI, 2.00-2.54] vs 2.63 [95% CI, 2.36-2.91] correct responses; *P* < .001) (eTable 8 in Supplement 2). Poisson regression showed a nonsignificant 5% lower rate of correct responses in the intervention group (IRR, 0.95 [95% CI, 0.90-1.00]; *P* = .07). There was no evidence of overdispersion (α ~ 0; likelihood-ratio test of α = 0: *P* = .48), and negative-binomial regression produced identical estimates of effect size and similar CIs to the Poisson model. Higher baseline knowledge (IRR, 1.07 [95% CI, 1.06-1.08]; *P* < .001), older age (IRR, 1.002 [95% CI, 1.00-1.00]; *P* = .04), and identifying as a woman (IRR, 1.07 [95% CI, 1.01-1.13]; *P* = .03) predicted greater improvement (eTable 9 in Supplement 2).

#### Intention to Vaccinate

At baseline, most participants responded “no” (181 [23.5%]) or “not sure” (344 [44.7%]) to the question regarding their intention to vaccinate. Positive change occurred in 72 participants (19.3% [95% CI, 1.53%-2.33%]) in the intervention group vs 66 (16.7% [95% CI, 1.30%-2.04%]) in the comparator group. Logistic regression showed a nonsignificant 19% higher odds of positive change in the intervention group (AOR, 1.19 [95% CI, 0.80-1.76]; *P* = .40). Older age predicted slightly lower odds (AOR, 0.99 [95% CI, 0.97-1.00] per year; *P* = .04); gender was not significant (eTable 10 in Supplement 2).

#### Self-Reported Vaccine Uptake

Follow-up surveys were completed by 533 participants (69.3%) between February and July 2025. Among these, 185 (34.7%) had changed or canceled travel plans, leaving 348 travelers (65.3%) to JE risk destinations for uptake analysis. Participants included in the uptake analysis tended to have higher education, greater prior JE awareness, more previous JE vaccination, and longer planned trips compared with those excluded (eTable 11 in Supplement 2). JE vaccine uptake was 35.1% overall (intervention vs comparator: 69 of 161 [42.9%] vs 53 of 187 [28.3%]).

Vaccinated travelers (n = 122) were younger (mean [SD] age, 40.5 [12.5] vs 45.4 [14.6] years), were more likely to spend more than 50% of their trip in rural areas (40 [32.8%] vs 22 [9.7%]), and were more likely to have consulted a clinician to discuss their trip (105 [86.1%] vs 69 [30.5%]) compared with nonvaccinated travelers (n = 226 [includes 12 who were unsure of their vaccination status]). Uptake was lower among those reporting past travel to visit family and relatives (vaccinated vs unvaccinated travelers: 46 [37.7%] vs 121 [54.5%]) and prior travel to Asia (65 [53.3%] vs 195 [86.3%]) but did not differ by gender or destination. Among vaccinated travelers, common reasons included concern about personal JE risk (54 [44.3%]), wanting to do everything possible to reduce risk (41 [33.6%]), and clinician recommendation (32 [26.2%]). Among those not vaccinated (n = 214), the most frequent reasons were low perceived risk (106 [49.5%]), belief that risk was not high enough (59 [27.6%]), and confidence in avoiding mosquito bites (35 [16.4%]). Cost and adverse effect concerns were reported by less than 10% in either group (eTables 12-14 in Supplement 2).

In multivariable analysis, intervention participants had more than twice the odds of reporting uptake (AOR, 2.22 [95% CI, 1.36-3.61]; *P* = .001) (Table 3). Postintervention intention strongly predicted uptake (AOR, 3.81 [95% CI, 2.35-6.17]; *P* < .001). Each additional year of age reduced odds by 2% (AOR, 0.98 [95% CI, 0.96-0.99]; *P* = .01); gender was not significant. In an exploratory analysis (not prespecified), neither baseline nor postintervention decisional conflict scores were significant predictors when added separately to the model; however, greater reduction in decisional conflict was associated with higher odds of vaccine uptake (AOR, 1.04 [95% CI, 1.02-1.06] per point reduction; *P* < .001), equivalent to a 4% increase in odds for each point decrease in DCS scores.

**Table 3.** Multivariable Logistic Regression Analysis of Self-Reported JE Vaccine Uptake

Variable	JE vaccine uptake, AOR (95% CI)	*P* value
Group		
Comparator (online government resource) vs intervention (JEVaDA [reference])	2.22 (1.36-3.61)	.001
Covariate		
Postintervention intention to vaccinate	3.81 (2.35-6.17)	<.001
Age	0.98 (0.96-0.99)	.01
Gender (women compared with men)	0.87 (0.54-1.50)	.34

Abbreviations: AOR, adjusted odds ratio; JE, Japanese encephalitis; JEVaDA, Japanese encephalitis vaccine decision aid.

## Discussion

In this randomized clinical trial, the web-based JEVaDA was not associated with a further reduction in decisional conflict compared with an active comparator. Across both groups, mean postintervention DCS scores fell below the threshold associated with uncertainty or delay, and nearly one-quarter (23.5%) of participants reached scores associated with decision implementation.^34^ However, almost one-third of participant scores (30.9%) remained above the uncertainty threshold, underscoring persistent decisional needs and the value of follow-up with clinicians. The lack of a between-group difference likely reflects the high quality of both resources and the absence of a no-information control.

In contrast, the JEVaDA intervention was associated with more than twice the odds of pretravel JE vaccine uptake. This finding may reflect the decision aid’s values clarification component, which helps travelers apply information to their personal circumstances and align intentions with behavior.^25,38,39^ However, this finding should be interpreted cautiously: loss to follow-up for this outcome was substantial, and uptake may be overestimated if those who were vaccinated were more likely to respond. Even so, the pattern is consistent with evidence that decision aids influence more than knowledge alone—they help users personalize risk and make choices aligned with their values.^38,39^ Among those who declined vaccination, greater awareness that JE is a mosquito-borne disease highlights the importance of bite avoidance measures, suggesting decision aids may also encourage complementary protective behaviors.

JE vaccination decisions are inherently preference sensitive: the disease is rare but potentially severe, recommendations are conditional, and exposure risk varies with itinerary and cumulative travel. In this cohort, many participants reported prior travel to Asia and itineraries combining shorter stays, rural travel, or destinations with recent imported cases.^10,40^ Given that JE vaccines provide high efficacy and, for the live-attenuated vaccine (Imojev), long-term protection,^41,42^ decision aids can help travelers weigh these benefits alongside cost and personal risk tolerance, particularly when planning repeated or higher-risk travel. Decision aids can be used before, during, or after consultations and may also serve travelers who do not seek clinical advice.^24,25^ Linking the JEVaDA tool to online platforms such as that for the present study’s comparator tool could increase reach.

Decision aids already exist for several other vaccines and populations, including COVID-19 and measles, mumps, and rubella.^23^ Further development should focus on additional preference-sensitive contexts, such as rabies preexposure and yellow fever vaccination in older adult travelers. The codesign framework offers a model for adapting decision aids for other settings, and work is under way to extend the JEVaDA tool for other populations.^25^ Systematic reviews confirm that decision aids improve knowledge and reduce decisional conflict^21,23^; our findings extend this evidence to travel medicine.

### Strengths and Limitations

The strengths of this study include its pragmatic design and recruitment outside travel clinics, reflecting the reality that many travelers do not seek pretravel care.^43,44^ This enhances generalizability and aligns with the decision aid’s purpose: to support travelers to access clear information, clarify values, and prompt discussion with clinicians. Measuring decisional conflict immediately before and after viewing the resource minimized confounding and reduced attrition seen in prior studies.^45,46^ Assessing decisional conflict alongside knowledge, intention, and uptake offered complementary insight into both immediate understanding and subsequent behavior. The JEVaDA tool was rigorously codesigned, met international IPDAS quality criteria, and is included in the Ottawa Hospital Research Institute A to Z Inventory of Patient Decision Aids,^47^ ensuring balanced, evidence-based content and values clarification.^20^

This study has some limitations. Our sample was relatively well educated and health literate, which may align with the profile of some Australian travelers but may limit generalizability to populations with lower health literacy.^24^ As is common for vaccine information, readability scores for both resources exceeded recommended levels for public-facing materials.^48^ Website analytics captured only a subset of visits due to cookie blocking; although self-reported engagement and REDCap time stamps showed similar patterns across groups, incomplete capture could bias engagement comparisons. Given the high-quality comparator, the study may have been underpowered to detect small between-group differences in decisional conflict. Conditioning the uptake analysis on postrandomization events (follow-up completion and travel to a JE risk destination) may have introduced selection bias. Self-reported vaccination is subject to recall and social desirability biases, which could differ between groups and introduce some misclassification.^49^ Ability to pay was not directly measured, limiting our assessment of financial barriers. Linking data to the Australian Immunisation Register was considered but deemed impractical due to incomplete reporting and privacy constraints. Self-reported uptake generally overestimates true uptake when compared with medical records.^50^ Maintaining up-to-date content requires ongoing resourcing.

## Conclusions

In this randomized clinical trial, the JEVaDA tool did not reduce decisional conflict beyond an active comparator but was associated with higher vaccine uptake. Decision aids may enhance patient engagement and support clinicians in delivering personalized, evidence-based care as global travel and JE epidemiology evolve. By helping travelers clarify values, understand risk (including cumulative exposure), and weigh practical considerations, tools like JEVaDA can support informed, values-congruent choices.

## Notes

Supplement 1.
Trial Protocol and Statistical Analysis Plan

Supplement 2. **eFigure 1.** Values Clarification Exercise Scoring Framework **eTable 1.** Comparison of Information Provided by the Japanese Encephalitis Vaccine Decision Aid (JEVaDA) (Intervention) vs the Healthdirect Online Government Resource (Active Comparator) **eTable 2.** Knowledge Questions on Japanese Encephalitis (JE) **eTable 3.** Distribution of Vaccine Confidence Index (VCI) Responses by Domain and Study Arm **eTable 4.** Web Analytics for Japanese Encephalitis Vaccine Decision Aid (JEVaDA) Website **eFigure 2.** Waterfall Plot of Individual Changes in Decisional Conflict Scale (DCS) Scores Pre to Post Intervention **eTable 5.** Sensitivity Analysis of the Analysis of Covariance Model Predicting Postreview Decisional Conflict Scale (DCS) Scores After Excluding 6 Extreme Outliers Identified via QQ Plot **eTable 6.** Comparison of Pre- and Postintervention Decisional Conflict Scale (DCS) Scores and DCS Subscale Scores, by Group **eTable 7.** Summary of Analysis of Covariance Results for Postreview Decisional Conflict Scale (DCS) Subscale Scores **eTable 8.** Comparison of Pre and Post Knowledge Scores by Group **eTable 9.** Poisson Regression Analysis of Japanese Encephalitis Knowledge **eTable 10.** Logistic Regression Analysis of Intention to Vaccinate **eTable 11.** Baseline Characteristics of Participants Included vs Excluded From the Uptake Analysis, Stratified by Randomized Group **eTable 12.** Characteristics of Travelers by Japanese Encephalitis (JE) Vaccine Uptake Status **eTable 13.** Reasons Selected for Getting Vaccinated Among Travelers Who Reported Japanese Encephalitis (JE) Vaccine Uptake Before Travel (n = 122) **eTable 14.** Reasons Selected for Not Getting Vaccinated Among Travelers Who Reported No Japanese Encephalitis (JE) Vaccine Uptake Before Travel (n = 214)
eReferences

Supplement 3.
Data Sharing Statement
