# Supplementary material for: Japanese Encephalitis Vaccine Decision Aid for Travelers: A Randomized Clinical Trial
Source: JAMA Netw Open. 2026 Jun 1;9(6):e2615190. doi: 10.1001/jamanetworkopen.2026.15190 (PMC13227307; doi:10.1001/jamanetworkopen.2026.15190)
Supplement: Supplement 1. — Trial Protocol and Statistical Analysis Plan [file jamanetwopen-e2615190-s001.pdf]

STUDY PROTOCOL

Evaluation of a Japanese encephalitis Vaccine Decision Aid

Protocol number: 1

Version: 2

Date: 17.11.25

Authors:

Dr. Sarah L. McGuinness, Mr. Owen Eades

Confidential

This document is confidential and the property of the School of Public Health and Preventive Medicine, Monash University. No part of it may be transmitted, reproduced, published, or used without prior written authorisation from the institution.

Statement of compliance

This study will be conducted in compliance with all stipulations of this protocol, the conditions of the ethics committee approval, and the NHMRC National Statement on Ethical Conduct in Human Research (2023).

Trial registration

This trial was registered with the Australian New Zealand Clinical Trials Registry (ANZCTR) September 25 2024. Registration number: **ACTRN12624001176550**

Document history

Version Number and Date	Summary of changes
**Version 1: 01/10/2024**	Original protocol submitted with ethics application
**Version 2: 17/11/2025**	Updated to include full trial registration details and revised funding statement

TABLE OF CONTENTS

CONTENTS

Table of Contents ... 1
1. Study Synopsis ... 4
1.1. Executive Summary ... 4
1.2. Key Details ... 4
1.3. Study Sponsor ... 4
1.4. Study Contributors ... 5
1.5. Glossary of Abbreviations and Terms ... 7
2. Introduction/Background Information ... 7
3. Study Objectives ... 8
3.1. Hypothesis ... 8
3.2. Study Objectives ... 8
4. Study Design ... 9
4.1. Intervention Arm ... 9
4.2. Control Arm ... 10
4.3. Governance ... 10
5. Study Methodology ... 10
5.1. Recruitment ... 10
5.1.1. Selection Criteria ... 11
5.2. Randomisation ... 11
5.3. Details of Participant Involvement ... 11
5.4. Consent and Withdrawal ... 12
5.5. Reimbursement / Incentives ... 12
5.6. Risks to Participants ... 12
5.7. Outcome Measures ... 13
5.7.1. Primary Outcome ... 13
5.7.2. Secondary Outcomes ... 13
5.7.3. Other Measures ... 14
5.8. Data Collection ... 14
5.9. Data Management and Storage ... 14
5.10. Data Analysis ... 15
5.11. Sample Size ... 15

5.12. Timeline 15
6. Ethics and Dissemination 16
6.1. Potential Conflicts of Interest 16
6.2. Dissemination and Translation Plan 16
7. Appendix 1: International Patient Decision Aid Standards (IPDAS) Checklist[18] 17
8. Appendix 2: Consumer Representative Involvement Agreement 20
9. Appendix 3: Consumer Representative Privacy and Confidentiality Agreement 22
10. Appendix 4: Combined Survey Questionnaire (Pre-intervention, Post-intervention, Follow-up) 23
11. Appendix 5: Participant Explanatory Statement 43
References 46

1. Study Synopsis

1.1. Executive Summary

This study is part of the TRAvel VAccine Decision Aids for Decision-making (TRAVAID) Project, which is establishing a platform for the development of a suite of evidence-based travel vaccine decision aids for travel medicine. This protocol details a randomised controlled trial (RCT), that will measure the impact of a co-designed Japanese Encephalitis VAccine Decision Aid (JEVaDA) compared with standard practice (a fact sheet) on vaccine decision-making for Japanese encephalitis (JE) in a cohort of Australian travellers.

1.2. Key Details

**TITLE**	Evaluation of a Vaccine Decision Aid for Japanese Encephalitis (JE)
**SHORT TITLE**	JE Vaccine Decision Aid Trial
**OBJECTIVES**	The aim of this study is to assess the effectiveness of a co-designed Japanese Encephalitis Vaccine Decision Aid (JEVaDA) compared to standard practice on JE vaccine decision-making amongst Australian travellers.
**DESIGN**	Individually randomised, single-blinded, controlled trial
**OUTCOMES**	Our primary outcome is to evaluate whether the JE decision aid reduces decisional conflict (as measured by the decisional conflict scale) compared to standard practice.<br><br>Secondary outcomes include evaluating the impact of the decision aid on JE knowledge, intention to vaccinate and actual vaccine uptake.
**STUDY DURATION**	1 year
**NUMBER OF PARTICIPANTS**	500
**STUDY LOCATION**	Melbourne, Australia
**Funding**	National Health and Medical Research Council (2017229-RSP)

1.3. Study Sponsor

**Study Sponsor**	Infectious Diseases Epidemiology Unit<br>School of Public Health and Preventive Medicine (SPHPM)<br>Monash University

**Contact Name**	Dr Sarah McGuinness
**Address**	553 St Kilda Road, Melbourne, VIC, 3004

1.4. Study Contributors

Name	Summary of contribution
**Dr Sarah McGuinness**	**Role:** Primary Investigator<br><br>**Affiliations:** Lecturer, Infectious Diseases Epidemiology Unit, SPHPM, Monash University; Lead Consultant Physician, Travel Medicine Clinic, Alfred Health<br><br>**Expertise:** Dr McGuinness is an infectious diseases physician and early career researcher with expertise in travel medicine and mixed methods research. She is an emerging global leader in the field of travel medicine and chairs the Digital Communications Committee of the International Society of Travel Medicine (ISTM).<br><br>**Contribution:** Dr McGuinness will oversee and coordinate this project and will lead study design; collection, management, analysis and interpretation of data, writing of reports and manuscripts for submission to peer-reviewed journals<br><br>**Email:** sarah.mcguinness@monash.edu
**Prof Karin Leder**	**Role:** Investigator<br><br>**Affiliations:** Head, Infectious Diseases Epidemiology Unit, SPHPM Monash University; Head, Travel Medicine and Immigrant Health Services, Victorian Infectious Diseases Service, Royal Melbourne Hospital<br><br>**Expertise:** Prof Leder is an infectious diseases physician involved in diverse areas of clinical and public health infectious disease research. She is recognised global leader in travel medicine with leadership roles in international travel medicine networks and societies.<br><br>**Contribution:** Prof Leder will be a member of the project's Steering Group and will contribute to study design, data interpretation, writing of summary reports and manuscripts and dissemination.<br><br>**Email:** karin.leder@monash.edu
**Prof Allen Cheng**	**Role:** Primary Investigator<br><br>**Affiliations:** Professor, Infectious Diseases Epidemiology Unit, SPHPM,

	Monash University; Director, Infectious Diseases, Monash Health<br><br>**Expertise:** Prof Cheng is an infectious diseases physician with research and policy expertise in vaccination. He has previously been chair of the Advisory Committee on Vaccines and the Australian Technical Group on Immunisation (ATAGI) and remains a member of ATAGI.<br><br>**Contribution:** Prof Cheng will be a member of the project's Steering Group and will contribute to data analysis and interpretation and writing of summary reports and manuscripts.<br><br>**Email:** allen.cheng@monash.edu
**A/Prof Holly Seale**	**Role:** Primary Investigator<br><br>**Affiliations:** School of Population Health, University of New South Wales<br><br>**Expertise:** A/Prof Seale is an infectious diseases social scientist with over 17 years of experience in undertaking social science research relating to immunisation and other prevention strategies.<br><br>**Contribution:** A/Prof Seale will be a member of the project's Steering Group and will contribute to study design, data analysis and interpretation, writing of summary reports and manuscripts and dissemination.<br><br>**Email:** h.seale@unsw.edu.au
**Mr Owen Eades**	**Role:** Project Officer<br><br>**Affiliations:** Infectious Diseases Epidemiology Unit, SPHPM Monash University<br><br>**Expertise:** Mr Eades is a research officer with experience in medical research coordination and project management support.<br><br>**Contribution:** Mr Eades will support the project's Steering Group and will contribute to study design, data collection and management, writing of summary reports and manuscripts and dissemination.<br><br>**Email:** owen.eades@monash.edu
**Ms. Jen Morris**	**Role:** Consumer Representative<br><br>**Affiliations:** Not applicable<br><br>**Expertise:** Ms. Morris has a background in consumer advocacy roles and is a frequent traveller who lives with a chronic illness.<br><br>**Contribution:** Ms. Morris will represent consumer interests as a

	member of the Steering Group and contribute to study design, interpretation, writing of summary reports and manuscripts and dissemination.<br><br>**Email:** jen.m.morris@gmail.com

1.5. Glossary of Abbreviations and Terms

Abbreviation/term	Description
AMR	Antimicrobial resistance
COVID-19	Coronavirus Disease 2019
DCS	Decisional Conflict Scale
IPDAS	International Patient Decision Aids Standards
ISTM	The International Society of Travel Medicine
JE	Japanese encephalitis
MMR	Measles, mumps & rubella
RCT	Randomised controlled trial
REDCap	Research Electronic Data Capture
SPHPM	School of Public Health and Preventive Medicine
VCI	Vaccine Confidence Index
VPD	Vaccine preventable diseases

2. Introduction/Background Information

Travellers play a key role in the global spread of infections, and as Australia has now fully re-opened its borders following COVID-19 disruptions, travel-related infection acquisition and importation is increasing. Pre-travel vaccination is a key preventive intervention for vaccine-preventable diseases (VPDs), but comes at a cost; factors contributing to poor uptake include low risk perceptions and willingness (and capacity) to pay.[(1-3)] While the COVID-19 pandemic has drawn attention to VPDs, it has also led to increasingly polarised vaccine attitudes, highlighting the need for health communication strategies that can address vaccine misinformation and support vaccine-related decision-making.[(4)] Vaccine decision aids are evidence-based tools designed to provide patients with high-quality information on vaccine risks and benefits, help them clarify and communicate personal values, and guide them through the decision-making processes; they can also be used to support

shared-decision making with healthcare providers.[5] Decision aids for influenza and MMR vaccines have been shown to reduce decisional conflict and increase vaccine uptake, but to date no studies have reported on development or evaluation of decision aids for travel-related VPDs.[6]

Among travel-related VPDs, JE stands out as a disease for which improved vaccine communication resources are needed. The uptake of JE vaccine amongst travellers to endemic areas is particularly poor, noting that travel to JE-endemic regions (comprising many Asia-Pacific countries) is common among Australians.[7, 8] The unpredictability of JE epidemiology, its propensity to cause severe disease and death (including in short-term travellers)[9-11] and expansion to new geographic areas (including 2022 detection in four Australian states)[12] make a compelling case to consider more widespread vaccination, including for travellers to endemic areas. Currently available vaccines are safe and efficacious[13] but vaccine decision-making is complicated by the availability of two vaccine options with different schedules, costs and duration of protection. Typhoid is another important travel-related VPD for which vaccine uptake in travellers to endemic regions is low despite considerable risk.[14] With rates of antimicrobial resistance (AMR) amongst *S.* Typhi strains increasing,[15] particularly in Asia which is a common travel destination for Australians, prevention through vaccination is increasingly important. As with JE, multiple vaccine options, including live vaccines, are available for typhoid, increasing decision-making complexity.[16] Evidence-based decision aids for travel-related VPDs could potentially increase travellers' understanding of disease risks, empower them to make informed decisions, and enhance pre-travel vaccine uptake to reduce disease importation.

3. Study Objectives

3.1. Hypothesis

We hypothesise that a co-designed Japanese Encephalitis Vaccine Decision Aid (JEVaDA) will reduce decisional conflict regarding JE vaccination compared to usual practice. This reduction in decisional conflict is expected to help users make higher-quality decisions that better align with their values.

We also hypothesise that by facilitating more informed and value-aligned decisions, JEVaDA will increase both the intention to vaccinate (planning to receive the vaccine before travel) and actual vaccine uptake. Additionally, we expect that JEVaDA will enhance knowledge related to JE.

3.2. Study Objectives

Guided by the Ottawa Decision Support Framework (ODSF), the main goal of this research is to assess the impact of a decision aid (JEVaDA) on Australian travellers' decision-making related to Japanese encephalitis (JE) vaccines. JEVaDA, co-designed with input from consumers and healthcare providers, is an online tool that aims to facilitate informed decision-making by helping travellers understand and visualise the risks, benefits and trade-offs of different choices, relate personal values to available options, and make choices aligned with their values.

Our primary outcome is to evaluate whether the JE decision aid reduces decisional conflict (as measured by the decisional conflict scale) compared to standard practice.

Secondary outcomes include evaluating the impact of the decision aid on JE knowledge, intention to vaccinate and self-reported vaccine uptake.

4. Study Design

This study will use an individually randomised, single-blinded, controlled trial design. Prospective Australian overseas travellers will be recruited through a consumer panel provider to participate in the trial.

Participation in the trial will involve three steps, plus an optional follow up. Participants will first complete an initial (pre-intervention) survey (Step 1). They will then be randomly assigned to either the intervention or control group and receive a link to the corresponding materials based on their group assignment. Participants will be instructed to keep the REDCap survey open in a separate browser tab while reviewing the assigned materials (Step 2). After reviewing the materials, they will return to the REDCap browser to complete a post-intervention survey (Step 3). Participants who complete Step 1 but do not commence Step 3 will be sent up to two invitational reminders via email between 48-72hrs after completion of Step 1. Additionally, participants will be asked to consent to be contacted 3-6 months after the initial survey to complete an optional follow-up survey. This follow-up will investigate whether they undertook travel and received JE vaccine. Participants may opt out of this follow-up contact if they choose.

4.1. Intervention Arm

Participants randomised to the intervention arm will receive a link to the online Japanese encephalitis vaccine decision aid (JEVaDA) tool. This tool has been developed in accordance with International Patient Decision Aids Standards (IPDAS – Appendix 1)[17, 18] and designed through a comprehensive co-design and user-testing framework.

The JEVaDA tool features a self-guided, step-by-step approach divided into three main sections, with additional pages such as an FAQ. It provides detailed information on Japanese encephalitis, including its geographic distribution, modes of transmission, clinical features, severity and risk. It also covers preventive measures, including mosquito bite avoidance strategies and a comparison of available vaccines in Australia. Participants are encouraged to reflect on their personal circumstances and how their beliefs and values align with their intended decision, and are prompted to consult with their healthcare provider to discuss their options.

The tool includes visual aids and interactive elements designed to support decision-making, such as a side-by-side risk-benefit analysis graphic and a values clarification exercise. JEVaDA is intended as an educational resource and emphasises the importance of consulting with a trusted healthcare provider before making a vaccination decision.

A downloadable short-form PDF version of the decision aid is available on the website. This PDF version contains a condensed version of the online content and lacks the interactive features of the online tool.

The time participants spend using the decision aid will vary based on their engagement with the content. Based on pilot testing, we anticipate that most users will spend approximately 15 minutes or less with the tool.

4.2. Control Arm

Participants randomised to the control arm will receive a link to an online health information page about JE managed by Healthdirect Australia. As the national virtual public health information service, Healthdirect Australia collaborates with federal, state and territory governments to provide a range of evidence-based health resources. Their platform features over 2,000 evidence-based and clinically safe pages of information.

The Healthdirect Australia JE webpage provides information on JE, including its geographic distribution, modes of transmission, symptoms and severity. It is also covers preventive options, including mosquito bite avoidance and vaccines available in Australia.

The information is organised into a single, continuous webpage, with key details broken down into sub-section. The page includes tools for symptom checking and finding local health services, as well as accessibility features such as text enlargement, page-masking and text-to-speech capabilities.

A print to PDF function is available at the top of the page, and links to additional resources and support are also included. Users are informed that reading the page in full takes approximately 11-minutes.

4.3. Governance

This project will be overseen by a Steering Group including investigators listed in Section 1.4 of this protocol. Meetings will take place at regular monthly intervals and will be held via Zoom. Additional meetings will be arranged as necessary.

In addition, we will host at least one consumer representative position as a member of the Steering Group. The role and expectations of the consumer representative (including commitment to privacy and confidentiality) are outlined in Appendices 2 and 3.

5. Study Methodology

5.1. Recruitment

We will recruit participants through the Online Research Unit (ORU), Australia's leading data collection agency (https://www.theoru.com/). The ORU specialises in delivering high-quality, purpose-built consumer panels and manages a nationally representative panel of over 350,000 active Australian members, profiled across diverse demographics. The ORU distinguishes itself by conducting the majority of its recruitment through offline methods, such as telephone, mail and print, in addition to its online recruitment efforts. This approach includes a double opt-in process and mailing incentives to respondents' residential addresses, which serves as a robust validation tool

to confirm participants' residency in Australia and ensure authenticity. All surveys are de-identified, and no personal identifying information will be shared with the research team.

Using a random stratified sampling approach, the ORU will distribute a survey link to a targeted subset of their panel members who meet the initial eligibility criteria: being 18 years or older and currently residing in Australia. The survey link will be sent via their online platform. After accessing the link, panel members will be screened for an additional criterion: a stated intention to travel to an Asian country where JE is a risk within the next 6 months. A list of at-risk countries and a map will be provided for reference. Respondents will be sampled to ensure national representativeness based on age, sex and geographic location. The ORU will continue to invite additional respondents as necessary to achieve the required demographic balance, adjusting for any under-represented groups based on completion rates.

5.1.1. Selection Criteria

In order to be considered eligible to participate in this study, participants must be:

- Age 18 years or older
- Currently living in Australia
- Able to read and understand English
- Planning travel to a JE-endemic region within the next 6-months

5.2. Randomisation

Individually stratified randomisation will be used to allocate participants to either the intervention or control group. This process will ensure that groups are balanced according to baseline demographic characteristics collected from the pre-intervention survey, including age group, gender and geographic location (state or territory of residence). We will utilise the automated randomisation module in REDCap, with access requested from the Monash Helix team. To maintain blinding, a statistician independent of the central research team will build and upload the randomisation tables to REDCap. Participants will be randomly assigned to their respective groups based on their survey responses. Upon completion of the pre-intervention survey, participants will be directed to the materials corresponding to their assigned group based on the randomisation outcome.

5.3. Details of Participant Involvement

Participation in the trial involves three steps, all completed in a single web-based session (Figure 1):

- Step 1: Pre-intervention survey (approximately 7-10 minutes): all participants will complete the same survey
- Step 2: Review of assigned information (approximately 15 minutes): participants will be randomised to review either the JEVaDA tool (intervention) or the Healthdirect JE page (control)

Figure 1: Flowchart of Participant Involvement

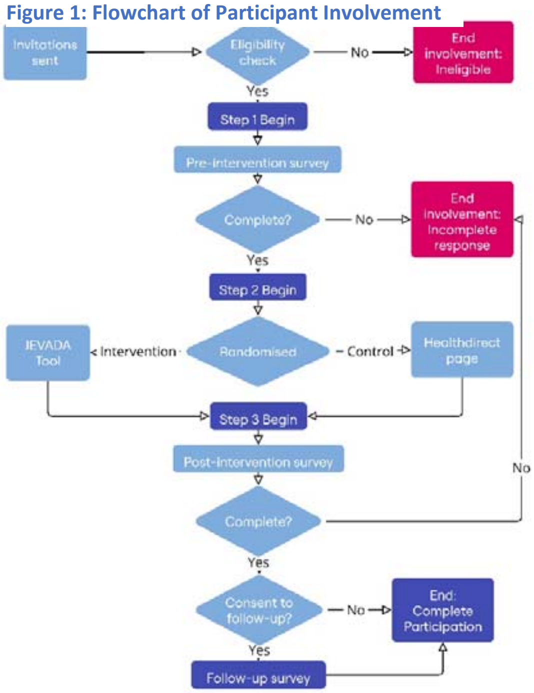

- Step 3: Post-intervention survey (approximately 5 minutes): all participants will complete the same survey

Participants who complete both the pre- and post-intervention surveys will be considered to have provided complete responses. The total estimated time commitment is up to 30 minutes.

Participants will also be asked to consent to a follow-up contact 3-6 months after the initial survey for an optional follow-up survey. This follow-up will assess whether they travelled and received the JE vaccine. Participants can choose to opt out of this follow-up contact. Those who do not opt out will be asked to complete a brief (5 minute) follow-up survey 3-6-months post-intervention.

Since this trial is conducted online, participants must have access to an internet connection in order to participate.

5.4. Consent and Withdrawal

Potential participants will be directed to an online REDCap survey via a Monash-hosted URL. The landing page will provide a brief overview of the study (see Appendix 4 for the landing page script) and a link to a PDF of the full Participant Explanatory Statement (Appendix 5).

Participants will see a consent statement that reads: "By clicking the link below, you consent to participate in this study". They will then have the option to click an "I Agree" button to begin the survey. Completion of the survey will imply consent to participate and permission to use the data collected for research purposes. Participants who decide not to take part may exit the survey at any time without any consequences.

5.5. Reimbursement / Incentives

The ORU incentivises survey completion using a point-cash system. Respondents earn points for completing surveys, with 1-point equating to $1. The number of points awarded is based on the length of the survey (i.e. the time spent completing it). The specific number of points awarded for this study will be determined by the ORU in accordance with their policies.

5.6. Risks to Participants

The anticipated risks to participants are minimal. Survey questions do not relate to sensitive topics. The survey will collect general demographic information, such as age, sex and place of residence (see Appendix 5: Pre-intervention survey – About you, p24). While this information might be categorised, it is not directly identifiable and is unlikely to lead to re-identification of individuals. Identifying details, such as names or dates of birth, are collected by ORU but will not be shared with study investigators and will not be part of the study data.

All survey data will be collected and managed within the REDCap database and stored in a secure, password-protected folder in password-protected electronic files on a secure Monash University Drive, accessible only to the core investigator team.

5.7. Outcome Measures

5.7.1. Primary Outcome

The Decisional Conflict Scale (DCS) will be used to measure decisional conflict. The DCS is a well-established tool in decision-making research and is frequently employed in studies evaluating the effect of patient decision aids.(19) We will use the traditional 16-item version of the DCS, which is psychometrically validated and assesses five dimensions of decision making. This version employs a statement format, which has been extensively tested and is the most commonly used version in the literature.

The DCS includes 16 statements, each rated on a 5-point Likert Scale with each response giving a point value: 0 = 'strongly agree', 1= 'agree'; 2 = 'neither agree nor disagree'; 3 = 'disagree'; 4 = 'strongly disagree'. Scores for each items are summed, divided by 16 and then multiplied by 25 to yield a total score ranging from 0 (indicating no decisional conflict) to 100 (indicating extremely high decisional conflict). Scores below 25 are typically associated with decision implementation, which scores above 37.5 are associated with delaying decisions or feeling unsure about decision-making.(20) Sub scores can be calculated across five dimensions (informed, values clarity, support, uncertainty and effective decision). The DCS exhibits good test-retest reliability and internal consistency, and correlates well with knowledge and decision discontinuance. It is responsive to change and discriminates between individuals who make decisions and those who delay.

While DCS scores may be sensitive to timing, there is no consensus on the optimal timing of measurement. Most decision aid studies assess DCS immediately or shortly after the intervention.(19) In this study, we will measure DCS scores immediately before and after the intervention to minimise confounding factors, such as seeking additional information, and to address follow-up attrition issues observed in previous vaccine decision aid studies that measured DCS scores 1 or 2 weeks post-intervention.(21, 22) The primary outcome will be the change in decisional conflict, as measured by the DCS, at an individual level for all participants, from pre- to post-intervention. The outcome will be assessed as the difference in the change in DCS scores (post- vs. pre-intervention) between the intervention group (who receive the decision aid) and the control group (who receive standard information).

5.7.2. Secondary Outcomes

Knowledge

Knowledge of JE will be assessed at the individual level by comparing participants' responses to 9 knowledge statements about JE in the pre- and post-intervention surveys. Participants will be asked to indicate whether they believe each statement true, false or if they are not sure. Responses will be categorised as correct (where participants correctly identify a true statement as true or a false statement as false), incorrect (where participants incorrectly identify a true statement as false or a false statement as true), or "not sure" responses. The secondary outcome will be the change in the proportion of correct responses from pre- to post-intervention, with the difference in knowledge gains compared between the intervention and control groups.

Similar to other decision aid evaluation studies,(21-23) these knowledge questions have been developed specifically for this study, based on the content presented in the decision aid, and

knowledge considered important in the literature.[24, 25] The statements have been reviewed and refined with input from both clinicians and consumers to ensure accuracy and relevance.

Intention to vaccinate

Intention to vaccinate will be assessed by comparing participants' responses to the question "Do you want to get a JE vaccine before your upcoming trip?" in both the pre- and post-intervention surveys. This question is based on the WHO's Behaviour and social drivers of vaccination survey priority questions.[26] Responses will be categorised into four groups: 1) those who don't want to get vaccinated, 2) those who are not sure, 3) those who intend to get vaccinated and 4) those who are already vaccinated. The primary outcome will by the change in the proportion of participants who express an intention to get vaccinated before their trip, comparing the intervention group to the control group.

Vaccine uptake

For participants who consent to the follow-up survey, uptake of JE vaccine will be assessed through self-reported responses to the question, " Did you receive a vaccine for Japanese encephalitis before your trip?". While this approach relies on self-reported data rather than clinical records, it is a pragmatic choice given the research team's capacity and the limitations of the Australian Immunisation Register, which does not require mandatory reporting of non-National Immunisation Program vaccines for adults. Additionally, it minimises participant burden and preserves anonymity.

5.7.3. Other Measures

The Vaccine Confidence Index (VCI) will be used to measure general attitudes towards vaccination. The VCI was developed by the Vaccine Confidence Project as a tool to explore perceptions towards the importance, safety, effectiveness and compatibility of vaccines with beliefs.[27] Consisting of 4-items measured on a 5-point Likert Scale, the VCI has been used to map global trends in vaccine confidence across 149 countries since 2015.[28] We will measure VCI scores as a baseline characteristic in the pre-intervention survey, prior to randomisation.

5.8. Data Collection

All data will be collected via online surveys using REDCap (Research Electronic Data Capture), a secure, web-based application designed to support data capture for research studies.[29] The surveys (Appendix 10) will gather information on participants' demographics, travel history, general health, health literacy, and JE vaccination history. Additionally, we will assess decisional conflict, knowledge, intention to vaccinate, and vaccine confidence as previously outlined. Questions from the WHO's Behavioural and Social Drivers (BeSD) survey will also be included to evaluate behavioural and social factors influencing vaccination decisions.[26] Furthermore, self-reported JE vaccine uptake data will be collected through an opt-out follow-up survey administered 6 months post-intervention.

5.9. Data Management and Storage

Survey data will be collected and managed using REDCap electronic data capture tools hosted and managed by Helix (Monash University). Monash University is subject to and ensures that personal and health information it holds or has access to, is handled in accordance with Australian legislation,

specifically the Privacy and Data Protection Act 2014 (Vic) and the Health Records Act 2001 (Vic). Data collected will be stored on University managed secure and resilient infrastructure located in Australia that complies with all applicable data protection and privacy obligations. All data will be accessible only to members of the Project Steering Group in a password-protected folder in password-protected electronic files. Data will be stored in accordance with the requirements of the Monash University Code for the Responsible Conduct of Research. Data will be retained at Monash University for at least seven years from the date of any publication which is based upon the data, and then destroyed by deletion of electronic files.

5.10. Data Analysis

Data will be analysed using the statistical analysis software package STATA v18. Baseline characteristics of participants will be tabulated by randomization groups. Continuous variables will be summarised using means (standard deviations), while categorical variables will be summarised by frequencies (percentages). Graphical visualisations will be conducted where appropriate.

Linear regression will be used to estimate the effect of intervention (vs control) on change in DCS scores (post- vs. pre-intervention) with pre-intervention DCS scores adjusted for in the model, i.e. an analysis of covariance (ANCOVA) structure. Statistical significance will be declared if a two-sided p-value is <0.05.

5.11. Sample Size

Sample size estimates are based on detecting an effect size of 0.30, as recommended in the DCS User Manual(30). Using the ANCOVA approach and assuming a modest correlation of 0.5 between pre and post-intervention DCS scores within-participant, we will have we will have over 90% power (2-sided significance level of 0.05) to detect an effect size of 0.30 in the primary outcome with a sample size of approximately 500 participants (assuming a 70% completion rate). The effect size is the difference in post-intervention DCS scores between intervention and control, adjusted for pre-intervention DCS score, and expressed as a fraction of the standard deviation of pre-intervention DCS scores.

5.12. Timeline

	**Month**						
**Activity**	1	2	3	4	5	6	7
Ethics approval & Trial Registration	X						
REDCap Project Build		X					
Recruitment			X				
Data collection			X				
Data review and analysis				X	X		
Write-up					X	X	
Dissemination						X	X

6. Ethics and Dissemination

This project will be reviewed by the Monash University Human Research Ethics Committee.

6.1. Potential Conflicts of Interest

Dr. Sarah McGuinness reports no conflict of interest relevant to this project. She receives fellowship funding from the NHMRC and royalties from her author roles for UpToDate. Prof. Karin Leder reports no conflict of interest relevant to this project. She receives fellowship funding from the NHMRC, Leadership consultancy from GeoSentinel, royalties from her author and editorial roles for UptoDate, and is an advisor for Travax. Prof Cheng receives funding from a NHMRC Investigator Grant and is a member of ATAGI, which makes recommendations on the clinical use of vaccines, including JE vaccines. The trial sponsor and funders have no role in the design, conduct, analysis and reporting of this trial.

6.2. Dissemination and Translation Plan

We will follow the Standards for Universal reporting of patient Decision Aid Evaluations (SUNDAE) guidelines when reporting results of this trial.[31] If the decision aid is found to be successful, an invitation to host an electronic version of the tool online will be offered to the International Society of Travel Medicine and/or the Australian-based National Centre for Immunisation Research and Surveillance. Results will also be submitted for publication in a scientific journal and may be presented at relevant conferences.

7. APPENDIX 1: INTERNATIONAL PATIENT DECISION AID STANDARDS (IPDAS) CHECKLIST[18]

**Content: Does the patient decision aid:**	
**Provide information about options in sufficient detail for decision making?**	
☐ Describe the health condition<br><br>☐ List the options<br><br>☐ List the option of doing nothing<br><br>☐ Describe the natural course without options<br><br>☐ Describe procedures<br><br>☐ Describe positive features (benefits)<br><br>☐ Describe negative features of options (harms, side effects, disadvantages)<br><br>☐ Include chances of positive / negative outcomes	**Additional items for tests:**<br><br>☐ Describe what test is designed to measure<br><br>☐ Include chances of true positive/negative and false positive/negative results<br><br>☐ Describe detection/treatment that would never have caused problems if one was not screened<br><br>☐ Describe possible next steps based on test result<br><br>Include chances the disease is found with/without screening
**Present probabilities of outcomes in an unbiased and understandable way?**	
☐ Use event rates specifying the population and time period<br><br>☐ Compare outcome probabilities using the same denominator, time period, scale<br><br>☐ Describe uncertainty around probabilities<br><br>☐ Use visual diagrams<br><br>☐ Use multiple methods to view probabilities (words, numbers, diagrams)	☐ Allows the patient to select a way of viewing probabilities (words, numbers, diagrams)<br><br>☐ Allow patient to view probabilities based on their own situation (e.g. age)<br><br>☐ Place probabilities in context of other events<br><br>☐ Use both positive and negative frames (e.g. showing both survival and death rates)
**Include methods for clarifying and expressing patients' values?**	
☐ Describe the procedures and outcomes to help patients imagine what it is like to experience their physical, emotional, social effects	☐ Ask patients to consider what positive and negative features matter most<br><br>☐ Suggest ways for patients to share what matters most with others
**Include structured guidance in deliberation and communication?**	
☐ Provide steps to make a decision<br><br>☐ Suggest ways to talk about the decision with a health professional	☐ Include tools (worksheet / question sheet) to discuss options with others
**Development process: Does the patient decision aid:**	
**Present information in a balanced manner?**	

☐ Able to compare positive / negative features of options	☐ Shows negative / positive features with equal detail (fonts, order, display of statistics)
**Have a systematic development process?**	
☐ Includes a developers' credentials / qualifications<br><br>☐ Finds out what users (patients, practitioners) need to discuss options<br><br>☐ Has peer review by patient / professional experts not involved in development and field testing<br><br>☐ Is field tested with users (patients facing the decision; practitioners presenting options)	The field test with users shows the patient decision aid is:<br><br>☐ Acceptable<br>☐ Balanced for undecided patients<br>☐ Understood by those with limited reading skills
**Use up to date scientific evidence that is cited in a reference section or technical document?**	
☐ Provides references to evidence used<br><br>☐ Report steps to find, appraise, summarise evidence<br><br>☐ Report date of last update<br><br>☐ Report how often patient decision aid is updated	☐ Describe quality of scientific evidence (including lack of evidence)<br><br>☐ Uses evidence from studies of patients similar to those of target audience
**Disclose conflicts of interest?**	
☐ Report source of funding to develop and distribute the patient decision aid	☐ Report whether authors or their affiliations stand to gain or lose by choices patients making after using the patient decision aid
**Use plain language?**	
☐ Is written at a level that can be understood by the majority of patients in the target group<br><br>☐ Is written at a grade 8 equivalent level or less according to a readability score (SMOG or FRY)	☐ Provides a way to patients understand information other than reading (audio, video, in-person discussion)
**Extra criteria if internet based:**	
☐ Provides a step-by-step way to move through the webpages<br><br>☐ Allow patients to search for keywords	☐ Provide feedback on personal health information that is entered into the patient decision aid
**Extra criteria if uses stories:**	
☐ Use stories that represent a range of positive and negative experiences<br><br>☐ Reports if there was a financial or other reason why patients decided to share their story	☐ State in an accessible document that the patient gave their informed consent to use their stories

**Effectiveness: Does the patient decision aid ensure decision making is informed and values based?**	
**Decision processes leading to decision quality. Does the aid help patients too:**	
☐ Recognise a decision needs to be made<br>☐ Know options and their features<br>☐ Understand that values affect decision	☐ Be clear about option features that matter most<br>☐ Discuss values with their practitioner<br>☐ Become involved in preferred ways
**Decision quality. Does the aid:**	
☐ Improves the match between the chosen option and the features that matter most to the informed patient	

8. Appendix 2: Consumer Representative Involvement Agreement

BACKGROUND

The TRAvel Vaccine AIds for Decision-making (TRAVAID) Project aims to develop evidence-based vaccine decision aids for travellers. We're using a mixed-methods approach to create decision aids for vaccine-preventable-diseases that involve complex decision-making processes. The success of this project hinges on tailoring these aids to user needs.

The role of TRAVAID Consumer Representatives is to contribute a consumer perspective at Steering Group meetings and advocate for consumer interests in the project's strategies and directions. Consumer Representatives are integral to this research, enhancing transparency and accountability. Your insights will ensure the research aligns with end-user perspectives and is relevant and impactful to the community.

Consumer Representatives are not employed or contracted by Monash University, however, as individuals involved and connected with the TRAVAID Project, we ask that you agree to certain conditions as outlined in this Agreement.

ROLES & RESPONSIBILITIES

The responsibilities of Consumer Representatives include to:

- Attend monthly Steering Group Meetings and promptly notify the research team if unable to attend
- Share lived experiences appropriately and effectively
- Respect the views of other Steering Group members and foster open communication.
- Actively engage in review of study materials, and provide feedback to ensure that materials are accessible, understandable, relevant and respectful of the diverse needs of the travel community.
- Inform the research team about potential conflicts of interest
- Contact the research team if you have concerns or unresolved issues about your role or collaboration

The responsibilities of the research team include to:

- Provide regular updates to Consumer Representatives involved in the TRAVAID Project as agreed upon at the commencement of their working partnership
- Provide notice in advance, when feasible, for document review requests and/or other ad-hoc activities
- Ensure that Consumers involved in the project understand its purpose, process and designated roles
- Acknowledge and respect the expertise and lived experience of Consumers involved in the research project.

CODE OF CONDUCT – BEHAVIOUR

Consumer representatives are expected to:

- Behave honestly and with integrity
- Act with due care and diligence
- Follow all applicable Australian laws
- Treat people equally and respectfully, regardless of culture, gender, age, or other features
- Encourage collaboration, communication and respect between members of the TRAVAID Steering Committee
- Participate in the activities outlined above

PAYMENT

Consumer Panel members are offered an 'honorarium' (payment) as a gesture of appreciation.

Honorarium amounts are as follows:

- $60 for each Steering Committee meeting participated in (for each meeting, of up to one hour in length; this is inclusive of any pre-meeting reading that is required).
- $50 per hour for related activities such as providing feedback and comments on provided topics/papers.

These amounts reflect recommended practice when engaging consumer representatives as partners or advisers in health research. Consumer representatives will be given the option to accept the honorarium, or they can decline it; and there is no obligation on the consumer representative for future meetings whether or not they accept an honorarium for earlier participation. Honorarium amounts will increase by 2.5% every year.

Consumer Representatives will be able to choose how they would prefer to receive their honorarium (direct to bank account or voucher).

The honorarium payments are not subject to withholding tax or superannuation as there is no employer/employee between Consumer Representatives of the Steering Committee and the University. However, the ATO has indicated that payments may be subject to income tax in the recipient's hands where there is a connection to the recipient's income-producing activities (such as working in the health profession). We suggest that Consumer Representatives obtain their own taxation advice with regard to these payments.

PRIVACY AND CONFIDENTIALITY

As a consumer representative, it's possible that you may see, hear or be given confidential information as part of your role. Please carefully read and complete the Privacy and Confidentiality Agreement for this study.

TERMS

Please complete and return this form **only** if you agree with the following statements:

- I am participating as a consumer representative and am not legally obligated (i.e. contracted) to Monash University, the TRAVAID Project or any associated programs in regard to involvement.
- I will choose how regularly and how much I participate in any TRAVAID or associated program activities that I am invited to attend.
- I acknowledge that my involvement can be ended at any time, either by me or Monash University.
- I will be offered an honorarium (i.e. payment) as a gesture of appreciation for participation. The payment will not create any employee/employer relationship with the University and as such the University has no withholding or superannuation obligations in respect to the payment. I can choose to either accept or decline this honorarium with no impact on my participation. I understand that in certain circumstances (i.e where there is a connection to my income-producing activities) that the payment may be considered taxable income and will seek my own advice on this matter.
- I am not a current student or staff member of Monash University and do not hold an adjunct appointment with the University.
- I am not entitled to an ABN as I am not carrying on an enterprise in Australia related to this research

DECLARATION

I, ____________________________, declare that I have read and understood the terms of this Agreement and that I have had the opportunity to seek answers to any questions in relation to my involvement in the proposed research activity. I acknowledge it is my responsibility to maintain confidentiality of all information that is not public domain. I accept and will adhere to the conditions of this agreement.

I would prefer to receive my honorarium as:

☐ A direct deposit into my bank account

☐ An electronic (i.e. digital) gift card

☐ I do not wish to accept an honorarium for my participation

Signature: ____________________________ Date: ________________

9. Appendix 3: Consumer Representative Privacy and Confidentiality Agreement

As a consumer representative, it is possible that you will hear, see or be given confidential information as part of your role. This information may include personal details about community members, their families or healthcare staff. It may also include details about future plans, projects or money matters.

In line with the Australian Privacy Act (1988), during your involvement (and after your involvement ends) all confidential information must be treated in the strictest confidence. You must not remove, destroy, share or discuss any personal information unless members of the TRAVAID Steering Committee confirms to you that it is acceptable and lawful to do so.

DECLARATION

- I understand that I should not discuss confidential information with anyone outside of the TRAVAID Steering Committee (including family and friends)
- I understand that all papers and records containing confidential information must not be copied or left in a way that unauthorized persons can obtain access to them and must be kept safely and securely when not being used

Name of Consumer Representative	Signature	Date
______________________	___________	______________

Name of person signing on behalf of the TRAVAID Steering Committee	Signature	Date
	________________________ ___________	______________

The TRAVAID Steering Committee confirms that your personal information will be kept secure and confidential at all times.

10. Appendix 4: Combined Survey Questionnaire (Pre-intervention, Post-intervention, Follow-up)

JE Decision Aid RCT Pre-intervention Survey

Landing page script

Thank you for your interest in this study run by Monash University.

What is this study about?

This study is about Japanese encephalitis (JE), a potentially serious disease that can affect travellers and residents in parts of Asia and Australia. While there are vaccines to prevent JE, few people get vaccinated. Deciding whether to get the vaccine can be hard because JE is rare, but it can cause severe illness or even death.

We're studying whether a decision aid could help travellers make informed choices about JE vaccination.

Who can participate?

Anyone currently living in Australia who plans to travel to a country in Asia where JE is a risk within the next 6 months.

What does participation involve?

Participation involves three steps, plus an optional follow-up:

- **Step 1: Initial survey** – You'll start by taking a short online survey (7-10 minutes) about yourself, your travel plans, what you know about JE, and your thoughts on JE vaccines
- **Step 2: Review information** – Next, you'll be randomly assigned to review either a general fact sheet on JE or the JE decision aid. Reviewing the online information may take about 15 minutes.
- **Step 3: Follow-up survey** – After reviewing the information, you'll take a short follow-up survey (about 5 minutes). This will help us see if your thoughts or decisions about JE vaccines have changed.
- **Optional follow-up** – We may contact you again in 3-6 months to ask if you received the JE vaccine before your trip. If you prefer not to be contacted again, you can let us know before submitting your responses.

What are the next steps?
Participation is entirely voluntary. For more details, please read the Participant Explanatory Statement <link to Appendix 5 PDF>.

If you choose not to participate, simply close your browser.

By clicking the link below, you consent to participate in this study.

**I agree – start the survey**

Questionnaire

(Screening): Travel intentions

These first questions are to find out if you're eligible for the study.

Japanese encephalitis (JE) is a risk in 24 countries, shown on the map and listed below.
NB: include a graphic of at-risk countries e.g. shaded map and country list

Are you planning to spend time in any of these countries (outside of Australia) in the next six (6) months? Only include countries where you're planning to leave the airport.

Yes	[continue survey]
No	[screen out]

If Yes: Please select the country where you plan to spend the most time from the drop-down list. If you will be spending equal time in more than one country, choose the country you will visit first. (required)
NB: drop-down menu limited to JE-endemic areas excluding Australia

About you

This next set of questions asks about you

Which State or Territory do you currently live in? (please select from the list)
NB: a drop-down list will be provided

What is your age (in years)? (*drop-down list: 18-100*)

What is your gender?

Female	☐
Male	☐
Non-binary/gender diverse	☐
My gender identity isn't listed. I identify as:	[text box]
Prefer not to say	☐

In which country were you born? (please select from the list)
NB: a drop-down list of all possible options will be provided, with Australia pinned at the top

Which country or countries were your parents or guardians born? (select all that apply)

- ☐ Australia
- ☐ China
- ☐ India
- ☐ Malaysia
- ☐ Philippines
- ☐ New Zealand
- ☐ South Africa
- ☐ United Kingdom (England, Scotland, Wales, Northern Ireland)
- ☐ Vietnam

☐ Other, please specify

Do you speak a language other than English at home?

No – English only	☐
Yes – Other, please specify	[text box]

Are you of Aboriginal or Torres Strait Islander origin?

No	☐
Yes, Aboriginal	☐
Yes, Torres Strait Islander	☐

For people of both Aboriginal and Torres Strait Islander origin, mark both 'Yes' boxes

What is the highest educational qualification you have completed?

Year 11 or below	☐
Year 12 or equivalent	☐
Certificate (e.g. TAFE or trade certificate)	☐
Undergraduate degree (e.g. Bachelor's degree)	☐
Postgraduate degree (e.g. Master's degree, PhD)	☐

What is your current employment status?

Employed full time	☐
Employed part time	☐
Employed casually/hourly	☐
In a family business without pay	☐
Home maker	☐
Student	☐
Unpaid voluntary work	☐
Unemployed	☐
Retired	☐

In general, would you say your health is?

Very good	☐
Good	☐
Moderate	☐
Poor	☐
Very poor	☐

Are you currently pregnant?

Yes	☐
No	☐

Not applicable	☐

Do you have any condition that weakens your immune system, or have you had any treatments (including medicines, chemotherapy or radiotherapy) in the past year that lower immune system function?

Yes	☐
No	☐

Please tell us which condition(s) or treatment(s) affect your immune system. Select all that apply:

- ☐ Leukaemia or lymphoma (blood cancer)
- ☐ Received an organ or stem cell transplant
- ☐ Living with HIV
- ☐ Taking steroids or immune-modulating medications (now or within last year)
- ☐ Receiving chemotherapy or radiotherapy (now or within last year)
- ☐ Other (please specify)

Health Literacy Assessment – the Single Item Literacy Screener

How often do you need to have someone help you when you read instructions, pamphlets, or other written material from your doctor or pharmacy?

- ☐ Never
- ☐ Rarely
- ☐ Sometimes
- ☐ Often
- ☐ Always

Travel experiences

You mentioned you'll be visiting a JE-risk area in the next 6 months. We'd now like to ask you some questions about your planned trip.

How many countries do you plan to visit during this trip?

- ☐ 1
- ☐ 2
- ☐ 3 or more

If one: Which country are you planning to visit during your upcoming trip? (Drop-down list – i.e. must select one only)
If two: What is the first country you are planning to visit during your upcoming trip? (Drop-down list)
If two: What is the second country you are planning to visit during your upcoming trip? (Drop-down list)
If three: What is the third country you are planning to visit during your upcoming trip? (Drop-down list)

When are you planning to start your trip?

- ☐ November, 2024
- ☐ December, 2024
- ☐ January, 2025
- ☐ February, 2025
- ☐ March, 2025
- ☐ April, 2025
- ☐ May, 2025

How long do you plan to travel for?

- ☐ less than one month
- ☐ 1 month or more

Where do you plan to spend the most time during your trip?

- ☐ More than half my time in cities
- ☐ More than half my time in rural areas (areas with a lot of farms or forests)
- ☐ About the same amount of time in both cities and rural areas
- ☐ Not sure

Are you planning to consult a healthcare provider for travel health advice for this trip?

- ☐ Yes, I'm planning to
- ☐ Yes, I've already done so
- ☐ No, I'm not planning to
- ☐ Not sure

If yes, where do you plan to get travel health advice from? (select all that apply)

- ☐ General practitioner / primary care
- ☐ Travel medicine clinic
- ☐ Pharmacist
- ☐ Internet
- ☐ Friend or relative
- ☐ Travel agent
- ☐ Other, please specify

This set of questions asks about your past travel experiences.

Have you ever travelled overseas (outside of Australia)?

- ☐ Yes
- ☐ No → *skip to "How many overseas trips…"*

In what year was your last overseas trip? _____ (Dropdown box: 2014 or earlier to 2024)

How many overseas trips have you taken in your lifetime? (select one only)

- ☐ 1 or 2
- ☐ 3 or 4
- ☐ Between 5 and 10
- ☐ More than 10
- ☐ None → *skip next 4 questions*

Which of the following regions have you visited in your lifetime? (select all that apply):

- ☐ Asia (excluding South Asia)

- ☐ South Asia (India, Pakistan, Bangladesh, Nepal, Sri Lanka)
- ☐ Africa
- ☐ North America
- ☐ Central and South America
- ☐ Europe
- ☐ Middle East
- ☐ Oceania (New Zealand, Papua New Guinea, Pacific Islands)
- ☐ Antarctica

NB: More than one response can be selected (checkboxes)

What were the reasons for your travel? (select all that apply)

- ☐ Tourism (holiday)
- ☐ Visiting friends and relatives
- ☐ Business (e.g. meeting, conference)
- ☐ Education (e.g. study abroad)
- ☐ Other, please specify____[text box]

Japanese encephalitis (JE)

This next set of questions relate to your knowledge of Japanese encephalitis.

Have you heard about JE before?

Yes	☐
No → *skip next question*	☐
Not sure → *skip next question*	☐

Where did you hear about JE before? (Select all that apply)

Internet	☐
General Practitioner (GP)	☐
Other healthcare provider (e.g. nurse, pharmacist, specialist, travel clinic)	☐
Family or Friends	☐
In the news/media (e.g. TV, radio, newspaper)	☐
Employer	☐
Community leader or religious leader	☐
This study	☐
Not sure	☐
Other, please specify:	☐

Please review each of the following statements about Japanese encephalitis (JE) and tell us if you think they are true, false, or if you're not sure:

Statements	True	False	Not sure
You can catch JE from another person		X	
JE is spread by mosquito bites	X		
JE can be serious: some people may die from it, and some who survive will have long-term health issues	X		
There is a specific treatment that can help your body fight JE		X	
JE can be prevented	X		
JE is caused by a bacteria		X	
Most travellers to JE risk areas don't get JE	X		
Two vaccines for JE are available in Australia	X		
Avoiding mosquito bites can lower your risk of getting JE	X		

NB: Correct answers are indicated with red crosses

How concerned are you about getting JE on your upcoming trip?

- ☐ Not at all concerned
- ☐ A little concerned
- ☐ Moderately concerned
- ☐ Very concerned

Vaccines in general - Vaccine Confidence Index

The next group of questions asks about how you feel about vaccines in general.
To what extent do you agree with the following statements?

	Strongly disagree	Tend to disagree	Tend to agree	Strongly agree	Do not know
Vaccines are important for people of all ages					
Vaccines are safe					
Vaccines are effective					
Vaccines are compatible with my beliefs					

Vaccination for Japanese encephalitis (JE)

This group of questions asks you specifically about JE vaccines.

Have you **ever** had a conversation with a healthcare professional about Japanese encephalitis (JE) vaccines?

Yes	☐
No	☐
Not sure	☐

Have you ever received a JE vaccine?

- ☐ No
- ☐ Yes → answer next two questions
- ☐ Not sure

If yes, Which JE vaccine did you receive?

- ☐ Imojev (live-attenuated)
- ☐ JEspect (inactivated) vaccine
- ☐ Not sure

If yes, How many doses did you receive?

- ☐ One dose
- ☐ Two doses
- ☐ Not sure

How important do you think getting a JE vaccine is for your health?

- ☐ Not at all important
- ☐ A little important
- ☐ Moderately important
- ☐ Very important

How safe do you think getting a JE vaccine is for you ?

- ☐ Not at all safe
- ☐ A little safe
- ☐ Moderately safe
- ☐ Very safe

How much do you trust the health workers who would give you a JE vaccine?

- ☐ Not at all
- ☐ A little
- ☐ Moderately
- ☐ Very much

Do you think most of your close family and friends would want you to get a JE vaccine?

- ☐ No
- ☐ Yes

Do you know where to go to get a JE vaccine for yourself?
- ☐ No
- ☐ Yes

How easy is it to pay for JE vaccination? When you think about the cost, please consider the cost of the vaccine (up to $300), would you say…
- ☐ Not at all easy
- ☐ A little easy
- ☐ Moderately easy
- ☐ Very easy

How easy is it for you to pay for other costs associated with JE vaccination? (e.g., any payments to the clinic, the cost of getting there and the cost of taking time away from work)
- ☐ Not at all easy
- ☐ A little easy
- ☐ Moderately easy
- ☐ Very easy

Do you want to get a JE vaccine before your upcoming trip?
- ☐ No, I do not want to
- ☐ Yes, I do want to
- ☐ I am not sure
- ☐ I am already vaccinated

Based on your choice for your upcoming trip, please answer the following questions:

	Strongly Agree	Agree	Neither Agree nor Disagree	Disagree	Strongly Disagree
I know which options are available to me					
I know the benefits of each option					
I know the risks and side effects of each option					
I am clear about which benefits matter most to me					
I am clear about which risks and side effects matter most to me					
I am clear about which is more important to me					

(the benefits or the risks and side effects)					
I have enough support from others to make a choice					
I am choosing without pressure from others					
I have enough advice to make a choice					
I am clear about the best choice for me					
I feel sure about what to choose					
This decision is easy for me to make					
I feel I have made an informed choice					
My decision shows what is important to me					
I expect to stick with my decision					
I am satisfied with my decision					

Pre-intervention exit page script

Important instructions for the Next Step

Now, we'd like you to review some information about JE and JE vaccines. You will be randomly assigned to review either a JE fact sheet or the JE decision aid. Please follow these steps:

1. **Click here** to open your assigned material in a new window.
2. **Keep this survey open in the current window** so you can easily return after your review
    a. If you're assigned the **fact sheet**, please scroll all the way to the bottom to access all the information
    b. If you're assigned the **decision aid**, please make sure you complete all four steps.
3. **Take your time to review the information at your own pace.** We estimate this will take around 10-15 minutes.
4. **Once you've finished, please return to this window to finish the survey**

Have you followed all of the steps above?

☐ Yes

Proceed

JE Decision Aid RCT Post-survey

Landing page script

Thank you for completing the initial survey and reviewing the resource you were assigned

What next?

We'd like to ask a few more questions to see if your thoughts about JE vaccines have changed. This follow-up survey is shorter and will take about 5 minutes to complete.

Please note that your answers will only be submitted when you finish the survey. You need to complete the survey to finalise your participation and receive your full reimbursement.

If you no longer wish to participate, simply close your browser. Your answers will not be saved.

Helpful tips

To prevent any issues, please the navigation buttons provided at the bottoms of the form to move between pages, rather than the browsers 'back' button.

If you are viewing the survey from a mobile device, we recommend adjusting your screen to landscape mode.

I agree – start the survey

Questionnaire

Decision Aid / Fact Sheet

This set of questions relates to your experience with the resource you were assigned to review

Which resource were you assigned to review?

- ☐ JE decision aid website – next five questions appear
- ☐ Health Direct JE fact sheet – next two questions only

Did you review the resource?

- ☐ Yes, the whole resource
- ☐ Yes, only part of it
- ☐ No, I didn't review it

Please estimate the amount of time you spent reviewing this information (in minutes): _____
NB: Text validation applied to ensure only whole numbers can be inputted

The length of the decision aid was:

- ☐ Too long
- ☐ Too short
- ☐ Just right

I found the decision aid was:

- ☐ Slanted towards alternatives to vaccination
- ☐ Slanted towards vaccination
- ☐ Balanced

The amount of information was:

- ☐ Too much
- ☐ Too little
- ☐ Just right

Please tell us your thoughts on the decision aid. What did you like, what didn't you like, and how can we improve it? _____ (free text, optional)

Japanese encephalitis (JE)

This next set of questions relate to your knowledge of Japanese encephalitis.

Please review each of the following statements about Japanese encephalitis (JE) and tell us if you think they are true, false, or if you're unsure:

Statements	True	False	Unsure
You can catch JE from another person		X	

JE is spread by mosquito bites	X		
JE can be serious: some people may die from it, and some who survive will have long-term health issues	X		
There is a specific treatment that can help your body fight JE		X	
JE can be prevented	X		
JE is caused by a bacteria		X	
Most travellers to JE risk areas don't get JE	X		
Two vaccines for JE are available in Australia	X		
Avoiding mosquito bites can lower your risk of getting JE	X		

NB: Correct answers are indicated with red crosses

How concerned are you about getting JE on your upcoming trip?

- ☐ Not at all concerned
- ☐ A little concerned
- ☐ Moderately concerned
- ☐ Very concerned

Vaccination for Japanese encephalitis (JE)

This group of questions asks you specifically about JE vaccines.

How important do you think getting a JE vaccine is for your health?

- ☐ Not at all important
- ☐ A little important
- ☐ Moderately important
- ☐ Very important

How safe do you think getting a JE vaccine is for you?

- ☐ Not at all safe
- ☐ A little safe
- ☐ Moderately safe
- ☐ Very safe

Do you think most of your close family and friends would want you to get a JE vaccine?

- ☐ No
- ☐ Yes

Do you know where to go to get a JE vaccine for yourself?

- ☐ No
- ☐ Yes

Do you want to get a JE vaccine before your upcoming trip?

- ☐ No, I do not want to
- ☐ Yes, I do want to

☐ I am not sure
☐ I am already vaccinated

If yes - Which vaccine option do you prefer?

☐ Imojev (live) vaccine
☐ JEspect (inactivated) vaccine
☐ I don't have strong feelings either way
☐ I want to get vaccinated but I haven't decided which option yet

Considering the option you prefer for your upcoming trip, please answer the following questions:

	Strongly Agree	Agree	Neither Agree nor Disagree	Disagree	Strongly Disagree
I know which options are available to me					
I know the benefits of each option					
I know the risks and side effects of each option					
I am clear about which benefits matter most to me					
I am clear about which risks and side effects matter most to me					
I am clear about which is more important to me (the benefits or the risks and side effects)					
I have enough support from others to make a choice					
I am choosing without pressure from others					
I have enough advice to make a choice					

I am clear about the best choice for me					
I feel sure about what to choose					
This decision is easy for me to make					
I feel I have made an informed choice					
My decision shows what is important to me					
I expect to stick with my decision					
I am satisfied with my decision					

Post-intervention end-page script

Thank you very much for participating. Your responses will help us understand if vaccine decision aids can help people making complex travel-related health choices

We're interested in contacting you again in 3-6 months to see whether this decision aid influences your behaviour while overseas.

Participation in this follow-up is completely optional. Please tell us whether you are happy to be contacted again in future below:

- ☐ I am happy to be contacted again
- ☐ I would prefer not to be contacted again

Please don't forget to press submit to save your survey responses

JE Decision Aid RCT Follow-up Survey

Landing page script

Thank you for participating in this study and agreeing to this follow-up survey.

What next?

We'd like to ask you a few questions about your recent trip to a country where Japanese encephalitis (JE) is a risk. We want to know whether you took any steps to protect yourself before and during your trip.

This survey should be quick to complete (less than 5 minutes).

Your answers are important, even if your plans changed and you didn't travel. They help us understand how to better support Australian travellers with vaccine decisions.

If you no longer wish to participate, simply close your browser. Your responses will not be saved. For more details, please read the Participant Explanatory Statement <Link to Appendix 5 PDF>.

I agree – start the survey

Questionnaire

In late 2024, you were planning a trip to a JE endemic area in [pipe previous answer]. Did you take your trip as planned?

- Yes – skip to 'please select the country you spent the most time in from the drop-down list'
- No

How did your plans change?

- Cancelled the trip
- Chose a different destination
- Delayed the trip
- Other (please specify)

Please provide any further details about why your travel plans changed (optional*). Free text box.* – end survey

Please review this list of countries before answering the next question:

☐ Bangladesh
☐ Bhutan
☐ Brunei Darussalem
☐ Burma (Myanmar)
☐ Cambodia
☐ China
☐ India
☐ Indonesia
☐ Japan
☐ Lao People's Democratic Republic
☐ Malaysia
☐ Nepal
☐ North Korea
☐ Pakistan
☐ Papua New Guinea
☐ Philippines
☐ Russia
☐ Singapore
☐ South Korea
☐ Sri Lanka
☐ Taiwan
☐ Thailand
☐ Timor-Leste
☐ Vietnam

Since November 2024, have you travelled to any of the countries listed above?

☐ Yes
☐ No – End survey

Please select the country you spent the most time in from the drop-down list (required)

NB: drop-down menu limited to JE-endemic areas excluding Australia

Did you visit a healthcare provider to discuss your trip before travelling overseas?

- Yes
- No – skip to 'Did you take any measures to avoid mosquito bites'

Did you receive a vaccine for Japanese encephalitis before your trip?

- Yes, I received the JEspect (inactivated) vaccine
- Yes, I received the Imojev (live-attenuated) vaccine
- Yes, but I don't recall which JE vaccine I received
- No, I did not get vaccinated – skip to 'Why did you decide not to get vaccinated'
- I don't remember

How many vaccine doses did you receive?

- 1 dose
- 2 doses (28 days apart)
- 2 doses (7 days apart)
- I don't remember

Why did you decide to get vaccinated? (Select all that apply)

- I was worried about my risk of catching JE
- I was worried about the lack of treatment options for JE
- I wanted to do everything I could to reduce my risk of JE
- My doctor recommended that I receive the vaccine
- I wasn't sure if I could avoid mosquito bites at all times
- I trusted that the JE vaccine is safe
- I believed that the JE vaccine would protect me
- The cost of the vaccine was not a concern for me
- Other, please specify

Why did you decide not to get vaccinated (Select all that apply)

- I wasn't worried about my risk of catching JE
- The lack of treatment options didn't worry me
- I didn't think the risk of JE was high enough
- My doctor did not recommend the vaccine
- My doctor told me I did not need the vaccine
- I was confident I could avoid mosquito bites
- I was worried about possible side effects
- I was not sure that the vaccine would protect me
- I was not able to pay the cost of the vaccine
- Other, please specify

Did you take any measures to avoid mosquito bites while overseas?

- Yes
- No

- Can’t recall

If yes, what measures did you take? (matrix-style question. Response options: frequently, sometimes, often)

- Wore long-sleeved shirts and long pants outdoors
- Applied insect repellent (e.g. containing DEET, picaridin or OLE) to exposed skin
- Stayed in screened rooms or under a permethrin-treated bed net
- Avoided going out after dark
- Other, please specify

How many times were you bitten by mosquitoes while overseas?

- Many times
- A few times
- Not at all

11. Appendix 5: Participant Explanatory Statement

Project ID: 44547
Project title: Evaluation of a Japanese encephalitis vaccine decision aid

Dr. Sarah L. McGuinness
School of Public Health and Preventive Medicine
Monash University
Chief Investigator
Phone: +61 3 9903 0118
Email: sarah.mcguinness@monash.edu

Thank you for your interest in this study. Please read all of this information carefully before deciding whether to participate. If you have any questions, you are welcome to contact the Chief Investigator.

Why are we doing this research?

This study is about Japanese encephalitis (JE), a serious disease that can affect travellers and residents in parts of Asia and Australia. While there are vaccines to prevent JE, not many people get vaccinated. Deciding whether to get the vaccine can be hard because JE is rare but can cause severe illness or death.

We are studying whether a decision aid - a tool designed to help people make informed health decisions - could assist Australians travelling overseas to make better choices about JE vaccination.

Who can participate?

Anyone living in Australia who plans to travel to a country in Asia where JE is a risk within the next 6 months.

What does participation involve?

Participation involves three steps and an optional follow-up:

- **Step 1: Initial survey** – You'll start by taking a short online survey (7-10 minutes) about yourself, your travel plans, what you know about JE, and your thoughts on vaccines
- **Step 2: Review information** – Next, you'll be randomly assigned to review either a general fact sheet on JE or the JE decision aid. This should take about 15 minutes.
- **Step 3: Follow-up survey** – After reviewing the information, you'll complete a short follow-up survey (about 5 minutes) to see if your views about JE vaccines have changed.
  Optional follow-up – We may contact you in 3-6 months to ask if you received the JE vaccine before your trip. You can opt out of this follow-up if you prefer.

Your participation is voluntary

Participation is completely voluntary and there is no cost to you. If you don't want to take part, simply close your browser. You can stop at any time, even after starting the survey, with no obligation to continue.

Please note that since this survey is anonymous, we **will not** be able to remove your responses once submitted.

Will I be compensated in any way for participating in the survey?

You will receive points in line with the ORU's reimbursement policy.

What are the benefits?

This research aims to help Australian travellers make informed decisions about JE vaccination. While you may not directly benefit, you'll have the chance to learn more about JE and contribute to research that could make travel safer.

What are the risks?

The materials in this study are educational and do not offer personalised medical advice. Always speak with your doctor before deciding on vaccination. The surveys do not cover sensitive topics or collect personal information. If you feel uncomfortable at any point, you are free to stop. If you or somebody you know has been affected by encephalitis, support is available from the Encephalitis Society (https://www.encephalitis.info/)

What will happen to my information?

This survey is anonymous. We will not collect any information that could identify you personally. All data will be stored securely on Monash University's password-protected servers. After publication, de-identified data may be shared publicly on Monash University's website (https://monash.figshare.com). It is possible that data from this study may also be used in other related research. Any shared data will only be in a non-identifiable form and accessed only where specific approval has been granted. All data will be stored electronically and held securely for 5 years and then deleted.

Results

The findings from this study will be shared in a research report. If the decision aid is helpful, it will be made available to the public on a Monash University website. Results will also be published in scientific journals and presented at conferences.

Source of funding

This research is supported by funding from the National Health and Medical Research Council (NHMRC) and Monash University.

Complaints

Should you have any concerns or complaints about the conduct of the project, you are welcome to contact the Executive Officer, Monash University Human Research Ethics Committee (MUHREC), quoting the following project ID: 44547

Executive Officer
Monash University Human Research Ethics Committee (MUHREC)
Room 111, Chancellery Building D,
26 Sports Walk, Clayton Campus
Research Office
Monash University VIC 3800

Tel: +61 3 9905 2052 Email: muhrec@monash.edu Fax: +61 3 9905 3831

Thank you,

Dr. Sarah L. McGuinness

References

1. Kain D, Findlater A, Lightfoot D, Maxim T, Kraemer MUG, Brady OJ, et al. Factors Affecting Pre-Travel Health Seeking Behaviour and Adherence to Pre-Travel Health Advice: A Systematic Review. J Travel Med. 2019;26(6).
2. McGuinness SL, Spelman T, Johnson DF, Leder K. Immediate recall of health issues discussed during a pre-travel consultation. J Travel Med. 2015;22(3):145-51.
3. Heywood AE, Zwar N. Improving access and provision of pre-travel healthcare for travellers visiting friends and relatives: a review of the evidence. J Travel Med. 2018;25(1).
4. Durand MA, Scalia P, Elwyn G. Can shared decision making address COVID-19 vaccine hesitancy? BMJ Evid Based Med. 2021.
5. Vujovich-Dunn C, Kaufman J, King C, Skinner SR, Wand H, Guy R, et al. A systematic review and meta-analysis of effectiveness of decision aids for vaccination decision-making. Vaccine. 2021;39(28):3655-65.
6. Scalia P, Durand MA, Elwyn G. Shared decision-making interventions: An overview and a meta-analysis of their impact on vaccine uptake. J Intern Med. 2021.
7. Mills DJ, Lau CL, Furuya-Kanamori L. Low uptake of Japanese encephalitis vaccination among Australian travellers. J Travel Med. 2021;28(3).
8. Duffy MR, Reed C, Edelson PJ, Blumensaadt S, Crocker K, Griggs A, et al. A survey of US travelers to Asia to assess compliance with recommendations for the use of Japanese encephalitis vaccine. J Travel Med. 2013;20(3):165-70.
9. Huang GKL, Tio SY, Caly L, Nicholson S, Thevarajan I, Papadakis G, et al. Prolonged Detection of Japanese Encephalitis Virus in Urine and Whole Blood in a Returned Short-term Traveler. Open Forum Infect Dis. 2017;4(4):ofx203.
10. Pyke AT, Choong K, Moore F, Schlebusch S, Taylor C, Hewitson G, et al. A Case of Japanese Encephalitis with a Fatal Outcome in an Australian Who Traveled from Bali in 2019. Trop Med Infect Dis. 2020;5(3).
11. Van K, Korman TM, Nicholson S, Troutbeck R, Lister DM, Woolley I. Case Report: Japanese Encephalitis Associated with Chorioretinitis after Short-Term Travel to Bali, Indonesia. Am J Trop Med Hyg. 2020;103(4):1691-3.
12. Furuya-Kanamori L, Gyawali N, Mills DJ, Hugo LE, Devine GJ, Lau CL. The Emergence of Japanese Encephalitis in Australia and the Implications for a Vaccination Strategy. Trop Med Infect Dis. 2022;7(6).
13. Furuya-Kanamori L, Xu C, Doi SAR, Clark J, Wangdi K, Mills DJ, et al. Comparison of immunogenicity and safety of licensed Japanese encephalitis vaccines: A systematic review and network meta-analysis. Vaccine. 2021;39(32):4429-36.

14. Forster DP, Leder K. Typhoid fever in travellers: estimating the risk of acquisition by country. J Travel Med. 2021;28(8).
15. Dyson ZA, Klemm EJ, Palmer S, Dougan G. Antibiotic Resistance and Typhoid. Clin Infect Dis. 2019;68(Suppl 2):S165-S70.
16. Andrews JR, Baker S, Marks F, Alsan M, Garrett D, Gellin BG, et al. Typhoid conjugate vaccines: a new tool in the fight against antimicrobial resistance. Lancet Infect Dis. 2019;19(1):e26-e30.
17. Stacey D, Volk RJ. The International Patient Decision Aid Standards (IPDAS) Collaboration: Evidence Update 2.0. Med Decis Making. 2021;41(7):729-33.
18. Elwyn G, O'Connor A, Stacey D, Volk R, Edwards AG, Coulter A, et al. International Patient Decision Aids Standards (IPDAS) Collaboration. Developing a quality criteria framework for patient decision aid: online international Delphi consensus process. British Medical Journal. 2006;333(7565):417-9.
19. Garvelink MM, Boland L, Klein K, Nguyen DV, Menear M, Bekker HL, et al. Decisional Conflict Scale Use over 20 Years: The Anniversary Review. Medical Decision Making. 2019;39(4):301-14.
20. O'Connor AM. Validation of a decisional conflict scale. Med Decis Making. 1995;15(1):25-30.
21. Shourie S, Jackson C, Cheater FM, Bekker HL, Edlin R, Tubeuf S, et al. A cluster randomised controlled trial of a web based decision aid to support parents' decisions about their child's Measles Mumps and Rubella (MMR) vaccination. Vaccine. 2013;31(50):6003-10.
22. Jackson C, Cheater FM, Harrison W, Peacock R, Bekker H, West R, et al. Randomised cluster trial to support informed parental decision-making for the MMR vaccine. BMC Public Health. 2011;11(1):475.
23. Witteman HO, Chipenda Dansokho S, Exe N, Dupuis A, Provencher T, Zikmund-Fisher BJ. Risk Communication, Values Clarification, and Vaccination Decisions. Risk Analysis. 2015;35(10):1801-19.
24. Yadav S, Ahmad S. An assessment of community participation in control and prevention of Japanese encephalitis in rural Uttar Pradesh. Int J Med Sci Public Heal. 2017.
25. Ahmad A, Khan M, Malik S, Jamshed S, Gogoi L, Kalita M, et al. Community knowledge and attitude towards Japanese encephalitis in Darrang, India: a cross-sectional study. Annals of Tropical Medicine and Public Health. 2017;10(2).
26. Organization WH. Behavioural and social drivers of vaccination: tools and practical guidance for achieving high uptake. 2022.

27. Larson HJ, Schulz WS, Tucker JD, Smith DM. Measuring vaccine confidence: introducing a global vaccine confidence index. PLoS Curr. 2015;7.

28. de Figueiredo A, Simas C, Karafillakis E, Paterson P, Larson HJ. Mapping global trends in vaccine confidence and investigating barriers to vaccine uptake: a large-scale retrospective temporal modelling study. Lancet. 2020;396(10255):898-908.

29. Harris PA, Taylor R, Minor BL, Elliott V, Fernandez M, O'Neal L, et al. The REDCap consortium: Building an international community of software platform partners. Journal of Biomedical Informatics. 2019;95:103208.

30. O'Connor A. User Manual - Decisional Conflict Scale 1993 [updated 2010. Available from: www.ohri.ca/decisionaid.

31. Hoffman AS, Sepucha KR, Abhyankar P, Sheridan S, Bekker H, LeBlanc A, et al. Explanation and elaboration of the Standards for UNiversal reporting of patient Decision Aid Evaluations (SUNDAE) guidelines: examples of reporting SUNDAE items from patient decision aid evaluation literature. BMJ Qual Saf. 2018;27(5):389-412.

Japanese Encephalitis Vaccine Decision Aid (JEVaDA) for Travellers: a Randomised Clinical Trial

Final Statistical Analysis Plan

Plan prepared by:
Sarah L McGuinness, MBBS, BMedSci, MPH, PhD[1,2]; Allen Cheng, MBBS, MPH, MBiostat, PhD[1,3]

[1]School of Public Health and Preventive Medicine, Monash University, Melbourne, VIC, Australia
[2]Department of Infectious Diseases, Alfred Health, Melbourne, VIC, Australia
[3]Department of Infectious Diseases, Monash Health, Melbourne, VIC, Australia

Version and Date:
Version 3.1 – 13 November 2025
Final SAP prior to data analysis (Version 3 finalised on 3 March 2025; Version 3.1 includes minor editorial changes only).

Contents

Section 1: General information 2
Section 2: Statistical analysis 2
Section 3. Changes to the Statistical Analysis Plan 7
References: 7

Section 1: General information

1.1 Study design

This is a single-blinded randomised controlled trial (RCT) evaluating the impact of a web-based Japanese encephalitis vaccine decision aid (JEVaDA) on informed vaccine decision-making and vaccine uptake among Australian travellers, compared to a government-funded online information sheet (HealthDirect). Participants are not blinded to allocation, but investigators are.

1.2 General principles

Analysis and reporting of results will follow the CONSORT guidelines.[1] Baseline characteristics will be tabulated by intervention group (JEVaDA) and comparator group (HealthDirect JE fact sheet) using appropriate summary statistics. Data will be derived from those patients who meet inclusion criteria (currently living in Australia, aged 18 years or older, able to read and understand English and intend to travel to a country outside of Australia where JE is a risk within the next 6 months) and enrol in the study.

1.3 Study population

The intention-to-treat (ITT) population will consist of all participants who were randomised to receive either the intervention or comparator, regardless of whether they reviewed their allocated intervention or completed the pre- and post-surveys. The only exclusion will be participants who withdraw their consent by exiting the pre-intervention survey prior to the material allocation stage.

The modified ITT population will consist of all participants who were randomised and provided both pre- and post-intervention data for the primary outcome.

Analyses for the primary and secondary outcomes will be conducted in the ITT and modified ITT populations, with the modified ITT analysis considered the principal analysis.

Section 2: Statistical analysis

2.1 Confidence intervals and p-values

For all statistical tests, a significance level of 5% will be employed (p value 0.05) and 95% confidence intervals will be reported.

2.2 Missing data

All available data will be used for the analysis. Multiple imputation will be used to handle any missing data.

2.3 Statistical software

All analyses will be conducted using StataBE 18.

2.4 Outcomes

The primary outcome will be post-intervention decisional conflict, as measured by the 16-item Decisional Conflict Scale (DCS)[2], with analyses adjusted for pre-intervention scores, age and sex. Secondary outcomes will include change in JE knowledge, change in intention to vaccinate and self-reported uptake of JE vaccine. Further details are included below.

2.5 Sample size analysis

Sample size estimates are based on detecting an effect size of 0.30 for the primary outcome, as recommended in the DCS User Manual [3]. Using the ANCOVA approach and assuming a modest correlation of 0.5 between pre and post-intervention DCS scores within-participant, we will have we will have over 90% power (2-sided significance level of 0.05) to detect an effect size of 0.30 in the primary outcome with a sample size of approximately 175 participants per group (total 350 patients). Target recruitment was set at 500 participants to account for a 30% non-completion rate. The effect size is the difference in post-intervention DCS scores between intervention and comparator, adjusted for pre-intervention DCS score, and expressed as a fraction of the standard deviation of pre-intervention DCS scores.

2.6 Primary Outcome (Decisional conflict)

Null hypothesis: There is no difference in post-intervention decisional conflict between Australian adults using an online JE vaccine decision aid (JEVaDA) and those using standard online health information (HealthDirect fact sheet) when planning overseas travel to JE risk areas.

Research question: Does use of an online JE vaccine decision aid (JEVaDA) lead to a greater reduction in decisional conflict compared to standard online health information (HealthDirect fact sheet) among Australian adults planning overseas travel to JE risk areas?

The primary outcome is post-intervention decisional conflict, measured using the 16-item Decisional Conflict Scale. Each item is rated on a 5-point Likert (0=strongly agree to 4=strongly disagree). The total score is calculated by summing all item scores, dividing by the number of items, and converting to a 0-100 scale. Higher scores indicate greater decisional conflict and a need for additional support or information. Scores below 25 are associated with being able to implement a decision, whereas scores above 37.5 are associated with decision delay or feeling unsure about implementation.

Mean DCS scores (with 95% confidence intervals) will be reported by group at baseline (pre-intervention) and post-intervention. Individual-level changes in score will be depicted in a waterfall plot, showing the change from baseline by group, with groups represented in different colours.

The primary analysis will use linear regression with an analysis of covariate (ANCOVA) structure where the post-intervention score is the dependent variable, the intervention group is the independent variable, and the pre-intervention DCS score, age and sex are included as covariates. Results will be presented in table format (Table 1). The table will include the estimated coefficients for each predictor (intervention group, baseline DCS score, age, sex) along with their 95% confidence intervals and p-values.

Table 1. Results of primary ANCOVA model predicting post-review DCS scores

Variable	Coefficient	95% CI	p-value
Intervention group (reference=JEVaDA)			
Baseline DCS			
Age (continuous)			
Female sex (vs. male)			
Non-binary (vs. male)			

The DCS has five subscales. Subscale scores will be presented in a similar way to the Total DCS score and will be considered secondary outcomes (Table 2).

Table 2. Decisional conflict scale and subscales: summary of statistical analyses to be performed

Analysis	Outcome	Groups compared	Included covariates	Test
Decisional conflict	Post-intervention DCS score	Intervention vs. comparator	Pre-intervention DCS score, age, sex	ANCOVA
Informed subscale	Post-intervention informed subscale DCS scores	Intervention vs. comparator	Pre-intervention informed subscale score, age, sex	ANCOVA
Values subscale	Post-intervention values subscale DCS scores	Intervention vs. comparator	Pre-intervention values subscale score, age, sex	ANCOVA
Support subscale	Post-intervention support subscale DCS scores	Intervention vs. comparator	Pre-intervention support subscale score, age, sex	ANCOVA
Uncertainty subscale	Post-intervention uncertainty subscale DCS scores	Intervention vs. comparator	Pre-intervention uncertainty subscale score, age, sex	ANCOVA
Effective decision subscale	Post-intervention effective decision subscale DCS scores	Intervention vs. comparator	Pre-intervention effective decision subscale score, age, sex	ANCOVA

2.7 Secondary outcomes

Secondary outcomes and planned statistical analyses are summarised in Table 3.

Table 3. Secondary outcomes: summary of statistical analyses to be performed

Analysis	Outcome	Groups compared	Adjusted factors*	Test
Knowledge of JE	Number of post-intervention correct responses to nine JE knowledge statements	Intervention vs. comparator	Pre-intervention knowledge score, age, sex	Poisson regression
Intention to vaccinate	Change in participants intention to get vaccinated	Intervention vs. comparator	Pre-intervention intent to get vaccinated, age, sex	Logistic regression
Uptake of JE vaccine	Self-reported pre-travel JE vaccine uptake	Intervention vs. comparator	Post-intervention intention to vaccinate, age, sex	Logistic regression

2.8. Secondary Outcome 1: JE knowledge

Null hypothesis: An online JE vaccine decision aid does not lead to a greater improvement in participants' knowledge of JE compared to standard online health information (HealthDirect fact sheet) among Australian adults planning overseas travel to JE risk areas.

Research question: Does use of JEVaDA lead to a greater increase in participants' knowledge of JE compared to standard online health information (HealthDirect fact sheet) among Australian adults planning overseas travel to JE risk areas?

Knowledge of JE will be assessed by the change in the proportion of correct responses to a set of nine JE knowledge statements from pre- to post-intervention. Participants will respond to the same nine statements at both time points, indicating whether they believe each statement is true, false or not sure (see Table 4). Responses will be coded numerically, and the total number of correct responses will be calculated for each participant at baseline and post intervention.

Table 4. JE knowledge statements

**Statements**	**True**	**False**	**Not sure**
You can catch JE from another person		X	
JE is spread by mosquito bites	X		
JE can be serious: some people may die from it, and some who survive will have long-term health issues	X		
There is a specific treatment that can help your body fight JE		X	
JE can be prevented	X		
JE is caused by a bacteria		X	
JE is a common disease in travellers to Asia		X	
Two vaccines for JE are available in Australia	X		
Avoiding mosquito bites can lower your risk of getting JE	X		

NB: Correct answers are indicated with red crosses

This secondary outcome will be analysed using Poisson regression. The dependent variable will be the post-intervention knowledge score (number of correct responses), with the number of questions included as an offset. The pre-intervention knowledge score (number of correct responses), along with age and sex will be included as covariates to adjust for baseline differences. Findings will be reported as incidence rate ratios (IRRs) with 95% confidence intervals (CIs) and p-values (Table 5).

Table 5. Poisson regression analysis of JE knowledge

Variable	Incidence rate ratio	95% CI	p-value
Intervention group (reference=JEVaDA)			
Pre-intervention knowledge score			
Age			
Female sex			

2.9 Secondary Outcome 2: Intention to vaccinate

Null hypothesis: The online JE vaccine decision aid (JEVaDA) does not lead to a greater increase in participants' intention to get vaccinated before their trip compared to standard online health information among Australian adults planning overseas travel to JE risk areas.

Research question: Does use of JEVaDA lead to a greater increase in participants' intention to get vaccinated before their trip compared to standard online health information among Australian adults planning overseas travel to JE risk areas?

Intention to vaccinate will be assessed by the change in response to the question "Do you want to get a JE vaccine before your upcoming trip?". This question will be asked in both the pre-intervention and post-intervention surveys. Response options include: "No, I do not want to", "Yes, I

do want to", "I am not sure", and "I am already vaccinated". For analysis, responses will be recoded into a binary variable, where "Yes, I do want to" is coded as 1, and all other responses are coded as 0. A new binary outcome variable will be created to capture positive change, defined as a shift from 0 pre-intervention to 1 post-intervention.

This outcome will be analysed using multivariable logistic regression. The dependent variable will be whether participants demonstrated a positive change in intention to vaccinate, and the primary independent variable will be group allocation. Baseline (pre-intervention) intention to vaccinate, age and sex will be included as covariates to adjust for baseline differences. Findings will be reported as adjusted odds ratios (ORs) with 95% confidence intervals (CIs) and p-values to indicate the strength and significance of the associations (Table 6).

Table 6. Logistic regression analysis of intention to vaccinate

Variable	Adjusted odds ratio (aOR)	95% CI	p-value
Intervention group (reference=JEVaDA)			
Baseline intention to vaccinate			
Age			
Female sex			

Secondary Outcome 3: JE vaccine uptake

Null hypothesis: The online JE vaccine decision aid (JEVaDA) does not lead to a greater increase in pre-travel JE vaccine uptake compared to standard online health information (HealthDirect fact sheet) among Australian adults planning overseas travel to JE risk areas.

Research question: Does use of JEVaDA lead to a greater increase in pre-travel JE vaccine uptake compared to standard online health information (HealthDirect fact sheet) among Australian adults planning overseas travel to JE risk areas?

JE vaccine uptake will be measured through self-reported responses to the question, "Did you receive a vaccine for Japanese encephalitis before your trip?" in an optional follow-up survey. Responses will be coded as 1 for "Yes" and 0 for "No" or "I don't remember".

This outcome will be analysed using multivariable logistic regression. The dependent variable will be self-reported JE vaccine uptake (binary) and the primary independent variable will be group allocation. As intention has been shown to be strongly associated with actual uptake and may confound the relationship between intervention and uptake, post-intervention intention to vaccinate will be included as a covariate, alongside age and sex. Findings will be reported as adjusted odds ratios (aORs) with 95% CIs and p-values (Table 7).

Table 7. Logistic regression analysis of JE vaccine uptake

Variable	Adjusted odds ratio (aOR)	95% CI	p-value
Intervention group (reference=JEVaDA)			
Post-intervention intention			

to vaccinate			
Age			
Female sex			

Section 3. Changes to the Statistical Analysis Plan

- Version 1 (3-Dec-2024): Included analyses for both intention-to-treat (ITT) and per-protocol populations. Primary outcome analysis specified as ANCOVA with change in DCS score as the dependent variable.
- Version 2 (19-Feb-2025): Revised to use a modified ITT population as the primary analysis set.
- Version 3 (3-Mar-2025): Revised primary outcome analysis to ANCOVA with post-intervention DCS score as the dependent variable, including pre-intervention DCS score as a covariate (rather than modelling change in scores). This approach was adopted to improve statistical efficiency and reduce bias associated with change-score models. This version represents the final SAP prior to data analysis.
- Version 3.1 (13-Nov-2025): Minor editorial changes to align with journal style requirements, including updates to the study title and section headings. No changes to planned analyses.

References:

1. Schulz KF, Altman DG, Moher D, Group C: CONSORT 2010 statement: updated guidelines for reporting parallel group randomised trials. BMJ 2010, 340:c332.
2. O’Connor AM. Validation of a Decisional Conflict Scale. Medical Decision Making. 1995 Feb 1;15(1):25–30.
3. O'Connor A. User Manual - Decisional Conflict Scale, 2010 update. Available from: www.ohri.ca/decisionaid.
