# Supplementary material for: Japanese Encephalitis Vaccine Decision Aid for Travelers: A Randomized Clinical Trial
Source: JAMA Netw Open. 2026 Jun 1;9(6):e2615190. doi: 10.1001/jamanetworkopen.2026.15190 (PMC13227307; doi:10.1001/jamanetworkopen.2026.15190)
Supplement: Supplement 2. — eFigure 1. Values Clarification Exercise Scoring Framework eTable 1. Comparison of Information Provided by the Japanese Encephalitis Vaccine Decision Aid (JEVaDA) (Intervention) vs the Healthdirect Online Government Resource (Active Comparator) eTable 2. Knowledge Questions on Japanese Encephalitis (JE) eTable 3. Distribution of Vaccine Confidence Index (VCI) Responses by Domain and Study Arm eTable 4. Web Analytics for Japanese Encephalitis Vaccine Decision Aid (JEVaDA) Website eFigure 2. Waterfall Plot of Individual Changes in Decisional Conflict Scale (DCS) Scores Pre to Post Intervention eTable 5. Sensitivity Analysis of the Analysis of Covariance Model Predicting Postreview Decisional Conflict Scale (DCS) Scores After Excluding 6 Extreme Outliers Identified via QQ Plot eTable 6. Comparison of Pre- and Postintervention Decisional Conflict Scale (DCS) Scores and DCS Subscale Scores, by Group eTable 7. Summary of Analysis of Covariance Results for Postreview Decisional Conflict Scale (DCS) Subscale Scores eTable 8. Comparison of Pre and Post Knowledge Scores by Group eTable 9. Poisson Regression Analysis of Japanese Encephalitis Knowledge eTable 10. Logistic Regression Analysis of Intention to Vaccinate eTable 11. Baseline Characteristics of Participants Included vs Excluded From the Uptake Analysis, Stratified by Randomized Group eTable 12. Characteristics of Travelers by Japanese Encephalitis (JE) Vaccine Uptake Status eTable 13. Reasons Selected for Getting Vaccinated Among Travelers Who Reported Japanese Encephalitis (JE) Vaccine Uptake Before Travel (n = 122) eTable 14. Reasons Selected for Not Getting Vaccinated Among Travelers Who Reported No Japanese Encephalitis (JE) Vaccine Uptake Before Travel (n = 214) eReferences [file jamanetwopen-e2615190-s002.pdf]

Supplementary Online Content

McGuinness SL, Eades O, Morris J, Seale H, Cheng AC, Leder K. Japanese encephalitis vaccine decision aid for travelers: a randomized clinical trial. *JAMA Netw Open*. 2026;9(6):e2615190. doi:10.1001/jamanetworkopen.2026.15190

eFigure 1. Values Clarification Exercise Scoring Framework

eTable 1. Comparison of Information Provided by the Japanese Encephalitis Vaccine Decision Aid (JEVaDA) (Intervention) vs the Healthdirect Online Government Resource (Active Comparator)

eTable 2. Knowledge Questions on Japanese Encephalitis (JE)

eTable 3. Distribution of Vaccine Confidence Index (VCI) Responses by Domain and Study Arm

eTable 4. Web Analytics for Japanese Encephalitis Vaccine Decision Aid (JEVaDA) Website

eFigure 2. Waterfall Plot of Individual Changes in Decisional Conflict Scale (DCS) Scores Pre to Post Intervention

eTable 5. Sensitivity Analysis of the Analysis of Covariance Model Predicting Postreview Decisional Conflict Scale (DCS) Scores After Excluding 6 Extreme Outliers Identified via QQ Plot

eTable 6. Comparison of Pre- and Postintervention Decisional Conflict Scale (DCS) Scores and DCS Subscale Scores, by Group

eTable 7. Summary of Analysis of Covariance Results for Postreview Decisional Conflict Scale (DCS) Subscale Scores

eTable 8. Comparison of Pre and Post Knowledge Scores by Groups

eTable 9. Poisson Regression Analysis of Japanese Encephalitis Knowledge

eTable 10. Logistic Regression Analysis of Intention to Vaccinate

eTable 11. Baseline Characteristics of Participants Included vs Excluded From the Uptake Analysis, Stratified by Randomized Group

eTable 12. Characteristics of Travelers by Japanese Encephalitis (JE) Vaccine Uptake Status[a]

eTable 13. Reasons Selected for Getting Vaccinated Among Travelers Who Reported Japanese Encephalitis (JE) Vaccine Uptake Before Travel (n = 122)

eTable 14. Reasons Selected for Not Getting Vaccinated Among Travelers Who Reported No Japanese Encephalitis (JE) Vaccine Uptake Before Travel (n = 214)

eReferences

This supplementary material has been provided by the authors to give readers additional information about their work.

© 2026 McGuinness SL et al. *JAMA Network Open*.

eFigure 1. Values Clarification Exercise Scoring Framework

	Strongly Disagree	Disagree	Unsure	Agree	Strongly Agree
I'm worried about my risk of JE	0	0	1	2	2
I'm worried that there's no treatment for JE	0	0	1	2	2
It's important to me to do everything I can to avoid getting JE	0	0	1	2	2
I'm sure that I can avoid moquito bites all the time	2	2	1	0	0
I'm worried about the side effects of the JE vaccine	2	2	1	0	0
I trust that the JE vaccine will protect me	0	0	1	2	2
I'm able to pay for the cost of vaccination	0	0	1	2	2

The values-clarification exercise (“What matters most to you?”,) asked participants to rate their agreement with seven statements on a 5-point Likert scale. Four items were positively framed (higher agreement indicating greater willingness to vaccinate), and three were negatively framed (higher agreement indicating greater hesitancy).

Responses were coded into three categories:

- Positively framed items: Strongly disagree/Disagree = 0; Unsure = 1; Agree/Strongly agree = 2
- Negatively framed items (reverse-coded): Strongly disagree/Disagree = 2; Unsure = 1; Agree/Strongly agree = 0

Scores were summed to yield a 0–14 summary score.

Thresholds for automated feedback were established and refined through piloting with consumers and clinicians. During piloting, participants reviewed hypothetical response patterns and evaluated whether the automated feedback (“leaning away,” “unsure,” “leaning towards”) aligned with their qualitative interpretation of the scenarios.

Final feedback categories:

- 0–4: Leaning away from vaccination
- 5–9: Undecided
- 10–14: Leaning towards vaccination

© 2026 McGuinness SL et al. *JAMA Network Open*.

eTable 1. Comparison of Information Provided by the Japanese Encephalitis Vaccine Decision Aid (JEVaDA) (Intervention) vs the Healthdirect Online Government Resource (Active Comparator)

**Information category**	**JEVaDA**	**Healthdirect online resource**
Japanese encephalitis (JE) disease information		
Definition	✓	✓
Transmission	✓	✓
Geographic distribution/epidemiology	✓	✓
Traveler risk	✓	✗
Likelihood of symptoms / clinical illness	✓	✓
Common symptoms	✓	✓
Serious complications	✓	✓
Treatment availability	✓	✓
Prevention strategies	✓	✓
Diagnosis	✗	✓
JE vaccine information		
Vaccines available in Australia	✓	✓
Trade names of vaccines available in Australia	✓	✗
How JE vaccines work	✓	✓
Type of vaccines (e.g. live-attenuated, inactivated)	✓	✗
Costs associated with vaccination	✓	✗
Vaccine side effects[a]	✓	✓
Likelihood of side effects	✓	✗
Duration of side effects	✓	✗
Dosage schedule	✓	~
Booster dose requirements	✓	✗
Administration method	✗	✓
Onset of protection	✓	✗
Duration of protection	✓	✗
Vaccine recommendations	✓	✓
Alternative options to vaccination		
Alternatives to vaccination	✓	✓
Effectiveness of alternative options	✓	✗
Decision-making prompts		
Consider destination risk[b]	✓	✗
Consider risks associated with itinerary	✓	✗
Consider personal health circumstances	✓	✗
Consider personal values (what matters most to them)[b]	✓	✗
Encouraged to speak with a healthcare provider	✓	✓
Record their beliefs or intentions[b]	✓	✗
Consider other travelers' experiences	✓	✗
Organisation/Presentation		
Information is chunked into short sections	✓	✓
Clear headers	✓	✓
Visual cues to key info (e.g. arrows, boxes, bullet points)	✓	✓
Visual aids (e.g. graphs, illustrations, infographics)	✓	✗
Tabulated information	✓	✓
At least one action is clearly identified	✓	✓

© 2026 McGuinness SL et al. *JAMA Network Open*.

Actions broken into manageable, explicit steps	✓	✗
Risks and benefits of actions articulated	✓	✗
The risks and benefits of different options compared	✓	✗
Interactive elements	✓	✓

[a] The comparator resource contained several generic hyperlinks (e.g., to general side-effect information) that were not specific to JE or vaccination and may have caused some confusion for users
[b] Interactive elements of the JEVaDA Tool
Legend: ✓ = Information provided; ✗ = not provided; ~ = Partial coverage
JEVaDA URL: https://www.monash.edu/medicine/sphpm/vaccinedecisionaids-je
Healthdirect JE URL: https://www.healthdirect.gov.au/japanese-encephalitis
Note: Comparison conducted in November 2024. The Health Direct webpage has since been updated.

© 2026 McGuinness SL et al. *JAMA Network Open*.

eTable 2. Knowledge Questions on Japanese Encephalitis (JE)[a]

**Statement**	**True**	**False**	**Not sure**
You can catch JE from another person		X	
JE is spread by mosquito bites	X		
JE can be serious: some people may die from it, and some who survive will have long-term health issues	X		
There is a specific treatment that can help your body fight JE		X	
JE can be prevented	X		
JE is caused by a bacteria		X	
Two vaccines for JE are available in Australia	X		
Avoiding mosquito bites can lower your risk of getting JE	X		

[a] Questions were adapted from previous knowledge, attitude and practice (KAP) studies[1,2] and refined by the research team, including input from a consumer representative. Items were worded to include a mix of true and false statements and ordered to avoid patterned responses. Participants reviewed each statement and indicated whether they believed it was true, false, or were not sure. Correct answers are marked with an X.

© 2026 McGuinness SL et al. *JAMA Network Open*.

eTable 3. Distribution of Vaccine Confidence Index (VCI) Responses by Domain and Study Arm

Domain	Response category	Comparator, n (%)	JEVaDA, n (%)	Total, n (%)	Overall % Agree[a]
Vaccines are important	Strongly agree	205 (51.8)	208 (55.8)	413 (53.7)	85.8
	Tend to agree	133 (33.6)	114 (30.6)	247 (32.1)	
	Tend to disagree	26 (6.6)	17 (4.6)	43 (5.6)	
	Strongly disagree	13 (3.3)	18 (4.8)	31 (4.0)	
	Don't know	19 (4.8)	16 (4.3)	35 (4.6)	
Vaccines are safe	Strongly agree	151 (38.1)	155 (41.6)	306 (39.8)	81.0
	Tend to agree	164 (41.4)	153 (41.0)	317 (41.2)	
	Tend to disagree	48 (12.1)	24 (6.4)	72 (9.4)	
	Strongly disagree	11 (2.8)	18 (4.8)	29 (3.8)	
	Don't know	22 (5.6)	23 (6.2)	45 (5.9)	
Vaccines are effective	Strongly agree	180 (45.5)	173 (46.4)	353 (45.9)	87.0
	Tend to agree	160 (40.4)	156 (41.8)	316 (41.1)	
	Tend to disagree	29 (7.3)	11 (3.0)	40 (5.2)	
	Strongly disagree	10 (2.5)	13 (3.5)	23 (3.0)	
	Don't know	17 (4.3)	20 (5.4)	37 (4.8)	
Vaccines are compatible with my beliefs	Strongly agree	198 (50)	196 (52.6)	394 (51.2)	84.9
	Tend to agree	137 (34.6)	122 (32.7)	259 (33.7)	
	Tend to disagree	26 (6.6)	19 (5.1)	45 (5.9)	
	Strongly disagree	14 (3.5)	18 (4.8)	43 (4.2)	
	Don't know	21 (5.3)	18 (4.8)	39 (5.1)	

[a] Proportion of participants who responded "Strongly agree" or "Tend to agree" within each domain

© 2026 McGuinness SL et al. *JAMA Network Open*.

eTable 4. Web Analytics for Japanese Encephalitis Vaccine Decision Aid (JEVaDA) Website[a]

	**Views (n)**	**Total users (n)**	**Average time on page (mm:ss)**	**Bounce rate[b] (%)**
**Individual webpage data**				
- Landing page	260	198	00:42	4.46%
- Step 1: Disease & vaccines	169	135	01:19	0.71%
- Step 2: Compare the risks & benefits	149	121	00:56	0.00%
- Step 3: Consider your situation	142	116	00:32	2.42%
- Step 4: Next steps	130	103	00:19	0.00%
- FAQ	32	30	00:54	0.00%
- Traveler Stories	31	26	00:33	6.67%
- About this decision aid	8	5	00:27	0.00%
- Home page	6	3	00:24	0.00%
- Resources	2	2	00:33	0.00%
**Country data**				
- Australia	873	191	02:48	6.64%
- Countries other than Australia	56	12	02:40	12.50%
**Source data**				
- Direct link	890	193	-	6.16%
- Other source (e.g., Google, Bing, Facebook)	39	10	-	6.25%
**Total Website Data ~**	929	203	02:47	-

[a] Data collected between 06 November 2024 and 10 November 2024. Data collected was heavily impacted by website visitor acceptance of the Monash cookie banner. It's estimated that presented data represents approximately 50% of the real visitor numbers. Data are reported at the page level for individual webpage data; otherwise, values represent site-wide totals. Average time data was not available for source data.
[b] Bounce rate refers to the percentage of website visitors who leave after viewing one page only, without further interaction with the site. Bounce rate was calculated only at the page level.

© 2026 McGuinness SL et al. *JAMA Network Open*.

eFigure 2. Waterfall Plot of Individual Changes in Decisional Conflict Scale (DCS) Scores Pre to Post Intervention

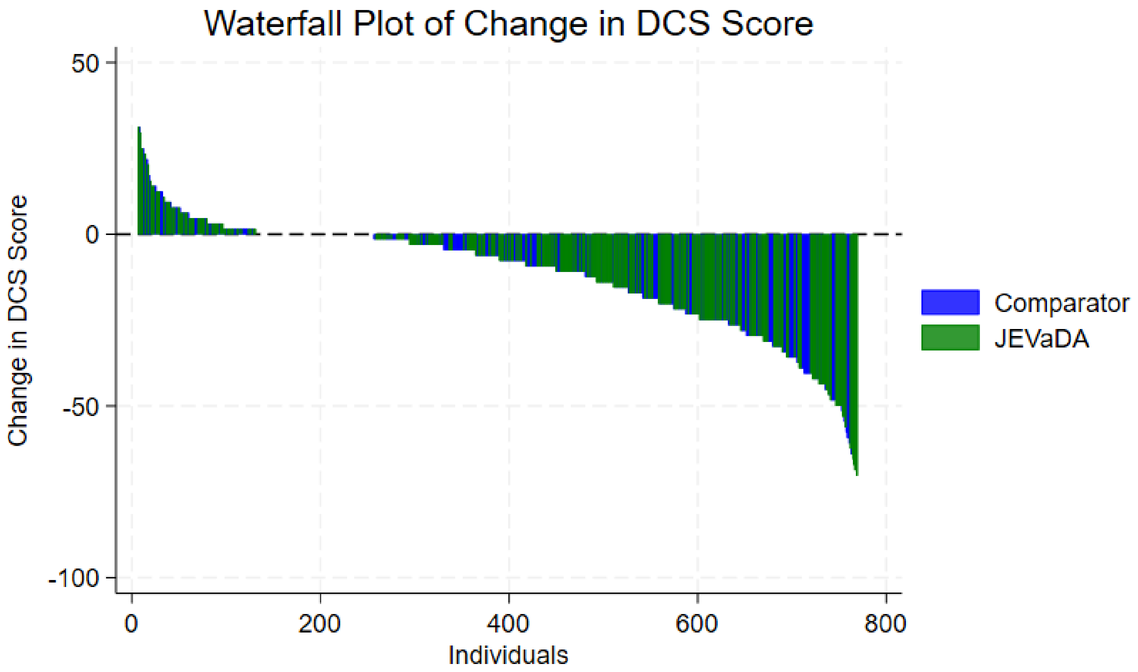

Each bar represents a participant's change in DCS score from pre- to post-intervention, ordered from largest increase to largest decrease. Negative values indicate reduced decisional conflict; positive values indicate increased conflict. Comparator group bars are blue; JEVaDA group bars are green.

© 2026 McGuinness SL et al. *JAMA Network Open*.

eTable 5. Sensitivity Analysis of the Analysis of Covariance Model Predicting Postreview Decisional Conflict Scale (DCS) Scores After Excluding 6 Extreme Outliers Identified via QQ Plot

Variable	Coefficient	95% CI	P value
Intervention Group (reference=JEVaDA)	-1.52	-3.44 to 0.41	.12
Baseline DCS	0.52	0.47 to 0.57	<.001
Age (continuous)	-0.12	-0.18 to -0.05	<.001
Woman (vs. man)	-1.64	-3.58 to -0.30	.10
Non-binary (vs. man)	3.78	15.03 to 20.39	.69

© 2026 McGuinness SL et al. *JAMA Network Open*.

eTable 6. Comparison of Pre- and Postintervention Decisional Conflict Scale (DCS) Scores and DCS Subscale Scores, by Group

Category	Group	Baseline mean score (95% CI)	Post-intervention mean score (95% CI)	Change in score (95% CI)	P value
DCS total score	Intervention (JEVaDA)	41.50 (39.41 to 43.59)	30.55 (28.76 to 32.35)	-10.94 (-12.81 to -9.07)	<.001
	Comparator (Health Direct)	44.45 (42.58 to 46.31)	32.87 (31.14 to 34.61)	-11.58 (-13.24 to -9.91)	<.001
DCS Informed Subscale	Intervention (JEVaDA)	46.29 (43.79 to 48.79	30.38 (28.46 to 32.31)	-15.91 (-18.58 to -13.24)	<.001
	Comparator (Health Direct)	48.42 (46.19 – 50.66)	32.51 (30.69 to 34.34)	-15.91 (-18.11 to -13.71)	<.001
DCS Values Clarity Subscale	Intervention (JEVaDA)	43.10 (40.55 to 45.65)	29.87 (27.88 to 31.86)	-13.23 (-15.70 to -10.76)	<.001
	Comparator (Health Direct)	45.81 (43.51 to 48.11)	32.32 (30.46 to 34.19)	-13.49 (-15.66 to -11.32)	<.001
DCS Support Subscale	Intervention (JEVaDA)	39.30 (37.10 to 41.50)	29.74 (27.81 to 31.66)	-9.56 (-11.48 to -7.64)	<.001
	Comparator (Health Direct)	41.94 (39.94 to 43.94)	31.86 (29.97 to 33.75)	-10.08 (-11.91 to -8.25)	<.001
DCS Effective Decision Subscale	Intervention (JEVaDA)	37.68 (35.51 to 39.86)	30.13 (28.18 to 32.08)	-7.56 (-9.50 to -5.61)	<.001
	Comparator (Health Direct)	41.32 (39.39 to 43.25)	32.58 (30.65 to 34.50)	-8.74 (-10.53 to -6.95)	<.001
DCS Uncertainty Subscale	Intervention (JEVaDA)	42.38 (39.94 to 44.82)	32.80 (30.78 to 34.81)	-9.58 (-11.75 to -7.41)	<.001
	Comparator (Health Direct)	45.79 (43.56 to 48.02)	35.19 (33.18 to 37.19)	-10.61 (-12.72 to -8.49)	<.001

© 2026 McGuinness SL et al. *JAMA Network Open*.

eTable 7. Summary of Analysis of Covariance Results for Postreview Decisional Conflict Scale (DCS) Subscale Scores

Subscale	Intervention Coefficient (95% CI)	P value	Baseline DCS Coefficient (95% CI)	P value	Age Coefficient (95% CI)	P value	Woman Coefficient (95% CI)	P value
**Informed**	–1.54 (–3.99 to 0.92)	.22	0.28 (0.23 to 0.33)	<.001	–0.16 (–0.24 to –0.08)	<.001	–1.87 (–4.35 to 0.62)	.14
**Values Clarity**	–1.51 (–3.92 to 0.90)	.22	0.35 (0.30 to 0.40)	<.001	–0.17 (–0.25 to –0.09)	<.001	–1.76 (–4.19 to 0.67)	.16
**Support**	–0.80 (–3.01 to 1.41)	.48	0.51 (0.45 to 0.56)	<.001	–0.14 (–0.22 to –0.07)	<.001	–1.20 (–3.43 to 1.02)	.29
**Effective Decision**	–0.57 (–2.83 to 1.70)	.62	0.52 (0.47 to 0.58)	<.001	–0.10 (–0.18 to –0.03)	.008	–0.56 (–2.84 to 1.72)	.63
**Uncertainty**	-0.85 (-3.29 to 1.58)	.49	0.45 (0.40 to 0.50)	<.001	–0.06 (–0.14 to 0.02)	.17	–1.40 (–3.86 to 1.05)	.26

© 2026 McGuinness SL et al. *JAMA Network Open*.

eTable 8. Comparison of Pre and Post Knowledge Scores by Group[a]

Group	Baseline mean knowledge score (95% CI)	Post-intervention mean knowledge score (95% CI)	Change in knowledge score (95% CI)	P value
Intervention	3.65 (3.38-3.91)	5.91 (5.66 – 6.17)	2.27 (2.00 – 2.54)	<.001
Comparator	3.55 (3.30-3.79)	6.18 (5.93 – 6.42)	2.63 (2.36 – 2.91)	<.001

[a]Knowledge score was calculated as the total number of correct responses out of 9 knowledge questions (see Supplementary Table S2). "Not sure" responses were coded as incorrect.

© 2026 McGuinness SL et al. *JAMA Network Open*.

eTable 9. Poisson Regression Analysis of Japanese Encephalitis Knowledge

Variable	Incidence rate ratio	95% CI	P value
**Group**			
Comparator vs. JEVaDA (reference)	0.95	0.90 – 1.00	.07
**Covariates**			
Pre-intervention knowledge score (prior knowledge)	1.07	1.06 – 1.08	<.001
Age	1.00	1.00 – 1.00	.04
Woman	1.07	1.01 – 1.13	.03

© 2026 McGuinness SL et al. *JAMA Network Open*.

eTable 10. Logistic Regression Analysis of Intention to Vaccinate

Variable	Odds ratio	95% CI	P value
**Group**			
Comparator vs. JEVaDA (reference)	1.19	0.80 – 1.76	.40
**Covariates**			
Age	0.99	0.97 – 1.00	.04
Woman	0.81	0.54 – 1.20	.29

© 2026 McGuinness SL et al. *JAMA Network Open*.

eTable 11. Baseline Characteristics of Participants Included vs Excluded From the Uptake Analysis, Stratified by Randomized Group

Characteristic	Comparator included (n=187)	Comparator excluded (n=222)	Intervention included (n=161)	Intervention excluded (n=244)
Age, mean (SD)	44.7 (14.7)	45.1 (15.62)	42.4 (13.1)	47.3 (16.7)
Gender, n (%)				
Woman	85 (45.5)	126 (56.8)	74 (46.0)	134 (54.9)
Man	102 (54.5)	95 (42.8)	87 (54.0)	109 (44.7)
Non-binary	0 (0.0)	1 (0.4)	0 (0.0)	1 (0.4)
Born in Australia, n (%)	102 (54.6)	138 (62.2)	106 (65.8)	162 (66.4)
Only English at home, n (%)	137 (73.3)	159 (71.6)	121 (75.2)	190 (77.9)
University degree	140 (74.9)	139 (62.6)	114 (70.9)	153 (62.7)
Health literacy: may need support, n (%)[a]	26 (13.9)	29 (13.1)	31 (19.3)	22 (9.0)
Main travel destination, n (%)				
Japan	31 (16.6)	49 (22.1)	32 (19.9)	61 (25.0)
Singapore	19 (10.2)	24 (10.8)	19 (11.8)	32 (13.1)
Indonesia	17 (9.1)	27 (12.2)	15 (9.3)	31 (12.7)
Thailand	14 (7.5)	21 (9.5)	12 (7.4)	22 (9.0)
China	20 (10.7)	19 (8.6)	16 (9.9)	15 (6.2)
India	19 (10.2)	17 (7.7)	15 (9.3)	16 (6.6)
Other	67 (35.8)	65 (29.3)	52 (32.3)	67 (27.5)
Trip duration ≤1 month, n (%)	49 (26.2)	37 (16.7)	43 (26.7)	48 (19.7)
Mostly urban travel, n (%)	111 (59.4)	138 (62.2)	101 (62.7)	147 (60.3)
Previous travel to Asia, n (%)	146 (78.1)	157 (70.7)	114 (70.8)	174 (71.3)
Previous VFR travel[b]	88 (47.1)	106 (47.8)	79 (49.1)	110 (45.1)
Heard of JE before, n (%)	71 (38.0)	71 (31.2)	65 (40.4)	75 (30.7)
Previous JE vaccination, n (%)	17 (10.2)	10 (4.5)	24 (14.9)	16 (6.6)

[a] Responded ‘sometimes’, ‘often’ or ‘always’ to the single-item literacy screener
[b] Asked only of participants who reported previous overseas travel

© 2026 McGuinness SL et al. *JAMA Network Open*.

eTable 12. Characteristics of Travelers by Japanese Encephalitis (JE) Vaccine Uptake Status[a]

Characteristic	Vaccinated (n=122)	Not vaccinated (n=226)[a]	Total (n=348)	P value
Group allocation				.005
JEVaDA	69 (56.6)	92 (40.7)	161 (46.3)	
Comparator	53 (43.4)	134 (59.3)	187 (53.7)	
Age, mean (SD)	40.5 (12.5)	45.4 (14.6)	43.7 (14.0)	.002
Gender, n (%)				.54
Woman	53 (43.4)	106 (46.9)	159 (45.7)	
Man	69 (56.6)	120 (53.1)	189 (54.3)	
Non-binary	0 (0.0)	0 (0.0)	0 (0.0)	
Trip duration ≥**1 month, n (%)**	37 (30.3)	55 (24.3)	92 (26.4)	.23
Spent >50% of time in rural areas	40 (32.8)	22 (9.7)	62 (17.8)	<.001
Main destination				.14
Japan	21 (17.2)	42 (18.6)	63 (18.1)	
Singapore	14 (11.5)	24 (10.6)	38 (10.9)	
Indonesia	7 (5.7)	25 (11.1)	32 (9.2)	
Thailand	7 (5.7)	19 (8.4)	26 (7.5)	
India	11 (9.0)	23 (10.2)	34 (9.8)	
Past VFR travel	46 (37.7)	121 (54.5)	167 (48.0)	.005
Past travel to Asia	65 (53.3)	195 (86.3)	260 (74.7)	<.001
Intention to vaccinate post-intervention	71 (58.2)	65 (28.8)	136 (39.1)	<.001
Visited healthcare provider to discuss trip[b]	105 (86.1)	69 (30.5)	174 (50.0)	<.001

[a]Includes 12 individuals who were unsure of vaccination status
[b]Question wording: "Did you visit a healthcare provider to discuss your trip before travelling overseas?". In Australia, all JE vaccines require administration by a healthcare provider; however, some vaccinated travelers may have answered "No" if they viewed their visit as solely for vaccination rather than for advice.
P values from two-sided t-tests (continuous) and Pearson x^2 (categorical)

© 2026 McGuinness SL et al. *JAMA Network Open*.

eTable 13. Reasons Selected for Getting Vaccinated Among Travelers Who Reported Japanese Encephalitis (JE) Vaccine Uptake Before Travel (n = 122)[a]

Reason	Comparator, n (%)	JEVaDA, n (%)	Total, n(%)
Worried about my risk of catching JE	24 (45.3)	30 (43.5)	54 (44.3)
Wanted to do everything I could to reduce my risk of JE	17 (32.1)	24 (34.8)	41 (33.6)
My doctor recommended that I receive the vaccine	14 (26.4)	18 (26.1)	32 (26.2)
Worried about the lack of treatment options for JE	13 (24.5)	11 (15.9)	24 (19.7)
I trusted that the JE vaccine is safe	14 (26.4)	7 (10.1)	21 (17.2)
Wasn't sure I could avoid mosquito bites all the time	10 (18.9)	6 (8.7)	16 (13.1)
I believed that the JE vaccine would protect me	7 (13.2)	9 (13.0)	16 (13.1)
The cost of the vaccine was not a concern for me	5 (9.4)	7 (10.1)	12 (9.8)
Other, please specify	1 (1.9)	0 (0.0)	1 (0.8)

[a]Participants who reported JE vaccine uptake before travel were asked: “Why did you decide to get vaccinated?” and presented with a predefined checklist of nine options. Multiple responses were allowed.

© 2026 McGuinness SL et al. *JAMA Network Open*.

eTable 14. Reasons Selected for Not Getting Vaccinated Among Travelers Who Reported No Japanese Encephalitis (JE) Vaccine Uptake Before Travel (n = 214)

Reason	Comparator, n (%)	JEVaDA, n (%)	Total, n(%)
I wasn't worried about my risk of catching JE	64 (51.6)	42 (46.7)	106 (49.5)
I didn't think the risk of JE was high enough	38 (30.6)	21 (23.3)	59 (27.6)
I was confident I could avoid mosquito bites	24 (19.4)	11 (12.2)	35 (16.4)
My doctor told me I did not need the vaccine	10 (8.1)	15 (16.7)	25 (11.7)
My doctor did not recommend the vaccine	12 (9.7)	10 (11.1)	22 (10.3)
I was worried about possible side effects	7 (5.6)	10 (11.1)	17 (7.9)
I was not sure that the vaccine would protect me	10 (8.1)	3 (3.3)	13 (6.1)
I was not able to pay the cost of the vaccine	7 (5.6)	3 (3.3)	10 (4.7)
The lack of treatment options didn't worry me	3 (2.4)	5 (5.6)	8 (3.7)
Other, please specify	3 (2.4)	5 (5.6)	8 (3.7)

Participants who reported no JE vaccine uptake before travel were asked: “Why did you decide not to get vaccinated?” and presented with a predefined checklist of ten options. Multiple responses were allowed. “Other” responses included “don’t like needles,” “short stay,” “it wasn’t high on my priority list,” and “I was already vaccinated.”

© 2026 McGuinness SL et al. *JAMA Network Open*.

eReferences

1. Kyaw PP, Shewade HD, Kyaw NTT, Hnin Phyo K, Lin HH, Kyaw AMM, Mya MM, Thaung S, Maung Maung YN. High vaccination coverage and inadequate knowledge: Findings from a community-based cross-sectional study on Japanese Encephalitis in Yangon, Myanmar. F1000Research 2020, 9:6 (https://doi.org/10.12688/f1000research.21702.2)
2. Yadav S, Ahmad S. An assessment of community participation in control and prevention of Japanese encephalitis in rural Uttar Pradesh. Int J Med Sci Public Heal. 2017 Nov 1. https://doi.org/10.5455/ijmsph.2017.0926622092017

© 2026 McGuinness SL et al. *JAMA Network Open*.
