# Supplementary material for: Japanese Encephalitis Vaccine Decision Aid for Travelers: A Randomized Clinical Trial
Source: JAMA Netw Open. 2026 Jun 1;9(6):e2615190. doi: 10.1001/jamanetworkopen.2026.15190 (PMC13227307; doi:10.1001/jamanetworkopen.2026.15190)
Supplement: Supplement 3. — Data Sharing Statement [file jamanetwopen-e2615190-s003.pdf]

# Data Sharing Statement

McGuinness. Japanese Encephalitis Vaccine Decision Aid for Travelers. *JAMA Netw Open*. Published June 01, 2026. doi:10.1001/jamanetworkopen.2026.15190

**Data**
**Additional Information:** Australian and New Zealand Clinical Trials Register (https://www.anzctr.org.au/): ACTRN12624001176550.
**Data available:** Yes
**Data types:** Deidentified participant data, Data dictionary
**How to access data:** The data that support the findings of this study are available from the corresponding author (SM: sarah.mcguinness@monash.edu), upon reasonable request
**When available:** With publication

**Supporting Documents**
**Document types:** None

**Additional Information**
**Who can access the data:** Researchers whose proposed use of the data has been approved
**Types of analyses:** Only for a specified purpose
**Mechanisms of data availability:** With investigator support
